# Design, Synthesis, and Evaluation of Selective Ubiquitin-Specific Protease 11 (USP11) Inhibitors

**DOI:** 10.1021/acsomega.5c08248

**Published:** 2025-12-05

**Authors:** Mostafa A. Hassan, Lodewijk V. Dekker, Michael J. Stocks, Ingrid Dreveny

**Affiliations:** † Biodiscovery Institute, School of Pharmacy, 6123 University of Nottingham, Nottingham NG7 2RD, United Kingdom; ‡ Department of Medicinal Chemistry, Faculty of Pharmacy, Assiut University, Assiut 71526, Egypt

## Abstract

Ubiquitin-specific protease 11 (USP11) is a promising anticancer target, but selective inhibitor development has proved to be challenging. Here, we designed and synthesized selective USP11 inhibitors based on a previously reported covalent probe (*N*-benzyl-4-(2-chloroacetyl)-1-methyl-1*H*-pyrrole-2-carboxamide, compound **2**) that is a more potent inhibitor of USP4 than USP11. Compound **7**, derived from **2** by the introduction of a piperidine moiety, exhibited an IC_50_ of 2.59 μM, while the replacement of chloroacetyl with a methyl sulfonylpiperazine moiety (compound **26**) displayed equivalent potency. Sustained inhibition of USP11 consistent with covalent binding was observed in a jump dilution assay, and the compounds displayed significantly lower activity against the paralogues USP4 and USP15. Both compounds inhibited the proliferation of PEO4 ovarian and MDA-MB-231 breast cancer cells. Notably, compound **26** displayed cytostatic activity, and normal fibroblast MRC-5 cells were less affected. The findings highlight the potential of **7** and **26** as promising candidates for the development of selective tool compounds and therapeutic agents targeting USP11.

## Graphical abstract

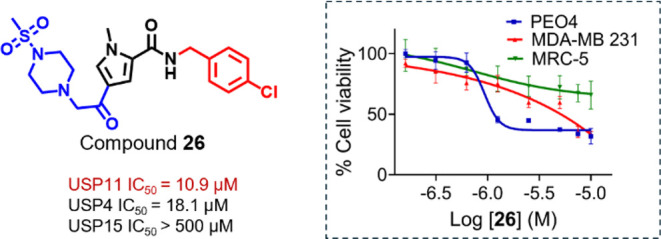

## Introduction

Protein ubiquitination is an essential post-translational modification that governs most cellular processes by targeting proteins for degradation or by modulating signaling pathways. It involves the covalent attachment of ubiquitin, a 76 amino acid polypeptide, to substrate proteins, thereby regulating their stability, localization, and activity.^1,2^ Ubiquitination can occur as monoubiquitination or polyubiquitination, where substrates are modified by either a single or by multiple ubiquitin additions.^3,4^ As such, this process is crucial for maintaining cellular homeostasis, controlling protein levels, and influencing key biological processes within cells.

Deubiquitinating enzymes (DUBs) are a class of proteases that cleave ubiquitin from substrate proteins, thereby reversing the biological consequences of ubiquitination.^5–7^

USP11, a member of the ubiquitin-specific proteases (USP) DUB subfamily is encoded by a gene located on chromosome Xp11.^8^ USP11 contains a cysteine protease domain with a catalytic triad that features the canonical USP core fold (Figure 1), which is essential for its enzymatic activity.^9^ Accordingly, the catalytic activity of USP11 depends on the catalytic triad cysteine residue, since mutation to serine results in the loss of its deubiquitinating function.^10^

**Figure 1:**
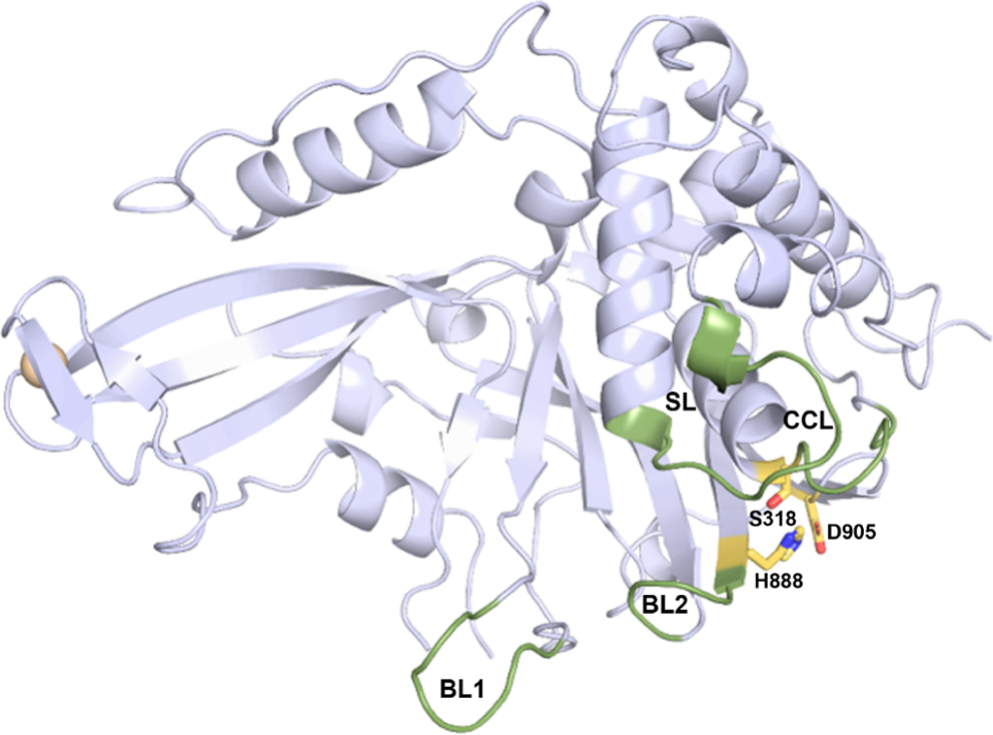
Cartoon representation of a USP11 catalytic domain crystal structure with a substrate mimetic present in the structure not shown; the yellow-colored amino acids are the catalytic triad residues where Cys318 has been mutated to a serine (PDB code: 8OYP^9^). The active site loops catalytic cleft loop (CCL), switching loop (SL), blocking loop 1 (BL1) and blocking loop 2 (BL2) are highlighted in green.

*Hs*USP11 and its paralogues USP4 and USP15 share ∼66% sequence identity in the catalytic domain and are often referred to as members of the DUSP-UBL (DU) subfamily of ubiquitin-specific proteases.^9–12^

Recent studies have implicated USP11 in the development and progression of several cancers, including breast, colorectal, ovarian, lung, and liver cancers, through regulating biological processes such as DNA repair, epithelial-mesenchymal transition, and cell cycle progression.^13–17^ USP11 regulates DNA double strand break repair by homologous recombination.^18^ In addition to its role in DNA repair, USP11 regulates the cell cycle and maintains protein stability of several other substrates by preventing proteasomal degradation through their deubiquitination.^15,19^ In breast cancer, USP11 has been associated with stabilizing BRCA2, a protein essential for repairing DNA double-strand breaks.^20^ Moreover, USP11 has been shown to enhance TGFβ-induced epithelial-mesenchymal transition, supporting the self-renewal of breast epithelial cells, and promoting tumor invasion and metastasis.^21^ In ERα-positive breast cancer patients, high USP11 levels correlate with lower survival rates, while low USP11 expression is associated with better prognosis.^22^ Similarly, USP11 promotes the epithelial-mesenchymal transition in ovarian cancer by deubiquitinating SNAIL.^23^ USP11 also enhances ovarian cancer chemoresistance by stabilizing BIP.^24^

These and other observations render USP11 an attractive therapeutic target in several cancers^13,25–27^ and USP11 inhibition could offer a novel approach for cancer treatment.^28,29^ However, while some compounds targeting USP11 have been reported (Figure 2), no inhibitors have entered clinical trials so far.^30,31^

**Figure 2:**
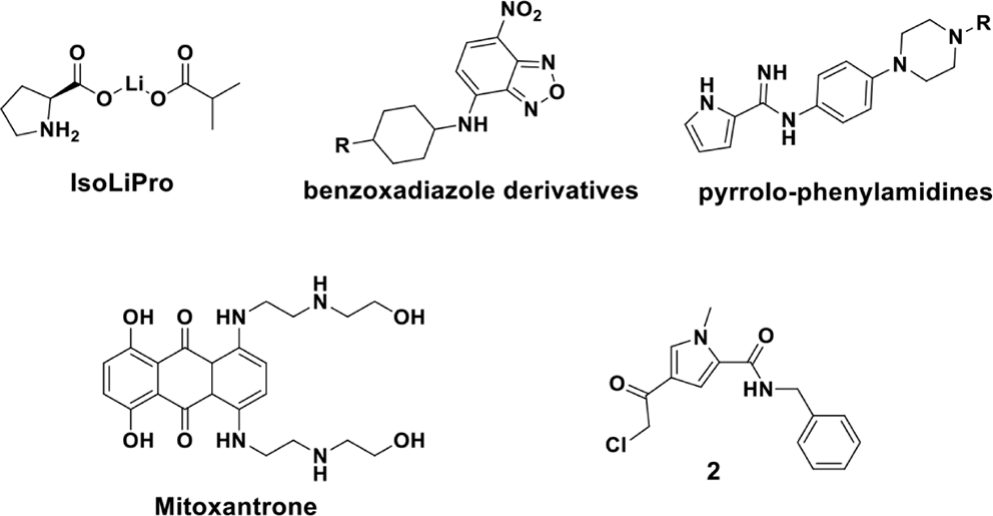
Structures of reported^33–36^ USP11 inhibitors.

In contrast to the multitargeted covalent DUB inhibitor PR619,^32^ IsoLiPro, a lithium isobutyrate-l-proline coordination compound (Figure 2), was reported as a small-molecule USP11 inhibitor that targets USP11 over USP25, and reduced tau and β-amyloid levels and improves cognitive function in Alzheimer’s disease models.^33^ Moreover, high-throughput virtual screening based on a USP11 homology model identified benzoxadiazole derivatives and pyrrolo-phenylamidine analogues (Figure 2) as two promising scaffolds.^34^ However, paralogue selectivity was not investigated.

Mitoxantrone (Figure 2), a topoisomerase, kinase and GTPase inhibitor,^37^ was identified as a USP11 inhibitor with an IC_50_ of 3.15 μM, but it is multitargeted highlighting the ongoing challenge of developing selective inhibitors.^35^ In 2016, Mission Therapeutics reported compound **2**,^36^ a warhead containing 4-chloroacetylpyrrole derivative, which demonstrated low micromolar inhibition of USP11 in human osteosarcoma cells.^36^ However, compound **2** was also a potent inhibitor of USP4, showing activity in the submicromolar range. In addition, compound **2** had limited plasma stability.^36^ Compound **2** has also been included into a library for the development of other DUB inhibitors.^38^

Given the role of USP11 in several malignancies and other diseases, there is a need to develop selective inhibitors targeting its catalytic domain in order to further probe USP11 function. The development of such inhibitors could lead to new therapeutic strategies for the treatment of multiple cancers, and other diseases, such as neurodegenerative diseases.^25,39,40^

Using compound **2** as a starting point for hit expansion, we report the discovery of novel compounds with enhanced selectivity for USP11 over its paralogues USP4 and USP15 while retaining its covalent interaction with USP11. Moreover, these compounds displayed promising behavior in cell-based assays. These findings highlight the potential of targeting USP11 with selective inhibitors as a strategy for cancer treatment.

## Results

### Design, Synthesis, and Biochemical Activity of USP11 Inhibitors

The starting point for the design of the compounds was based on the previously reported covalent compound **2**, which exhibited activity against USP11 within a low micromolar concentration range.^36^ To investigate whether potency could be increased a new series of amide derivatives was synthesized with altered substitutions at the benzyl ring system (**3–14**). Furthermore, the importance of the chloroacetyl moiety warhead was evaluated by synthesizing a derivative lacking this moiety (**16**) (Scheme 1).

**Scheme 1:**
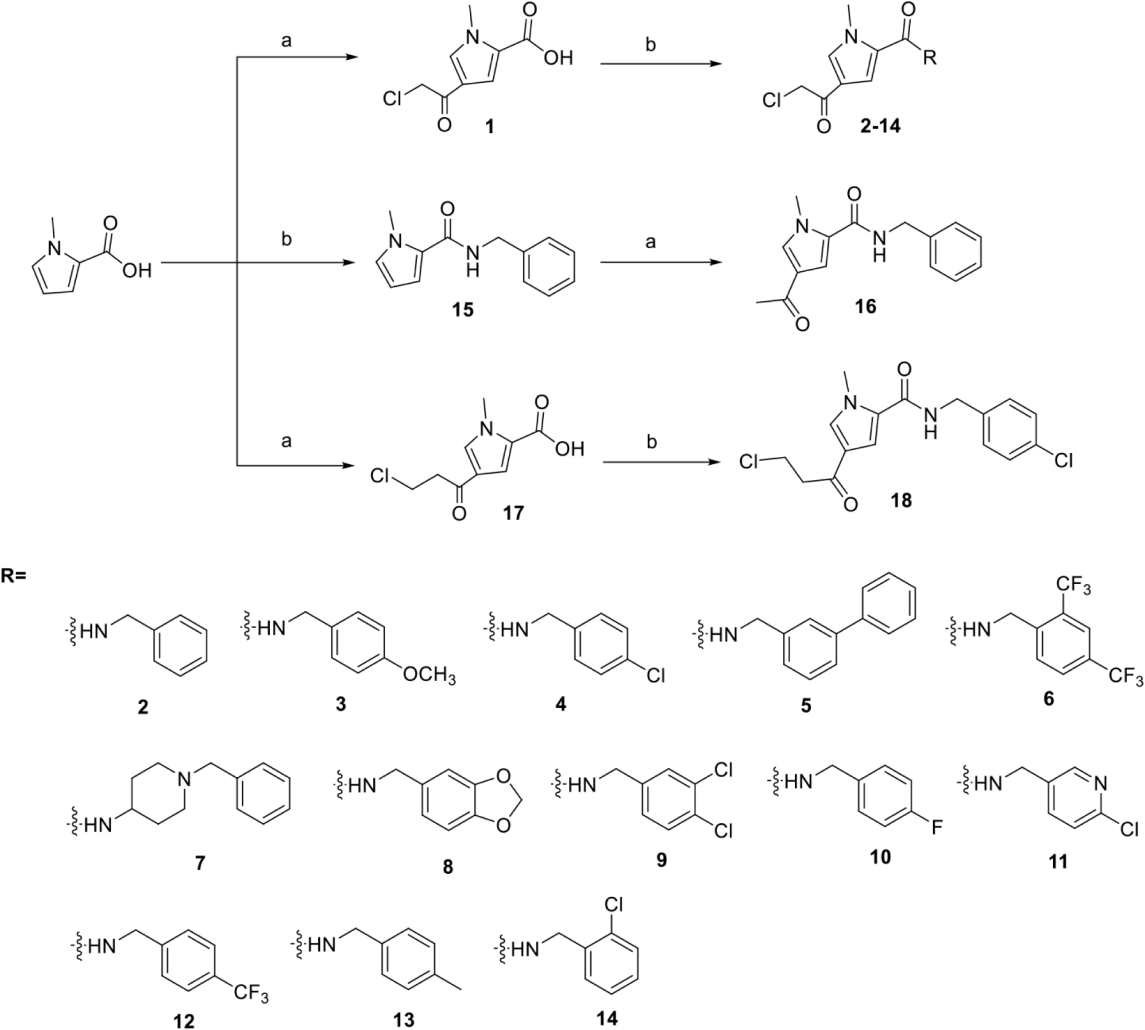
Synthesis of Compounds **2-–18** [Fn sch1-fn1]

i Reagents and conditions: (a) (i) 1-methyl-1*H*-pyrrole-2-carboxylic acid (1 equiv) or *N*-benzyl-1-methyl-1*H*-pyrrole-2-carboxamide (**15**), AlCl_3_ (2 equiv) in DCM 0 °C, 30 min. (ii) acyl chloride (1.1 equiv) 45 °C, 16 h. (iii) saturated NaHCO_3_ solution (100 mL), Celite filtered, conc. HCl, 40–52%. (b) (i) 1-methyl-1*H*-pyrrole-2-carboxylic acid or carboxylic acid derivatives **1** (1 equiv), EDC. HCl (1.2 equiv), HOBt (1.1 equiv), corresponding amine (1.2 equiv), THF, 16 h, rt. (ii) Saturated solution of citric acid (150 mL), ethyl acetate (100 mL) extraction, saturated sodium bicarbonate solution (100 mL), flash column chromatography for purification, 10–40%.

Building on the inhibitors **2–14**, which depend on an irreversible covalent warhead for USP11 binding, we aimed to investigate whether similar scaffolds could retain activity without relying on irreversible binding. To test this, a new series of analogues **19–24** was designed and synthesized in which the reactive chloroacetyl group was removed (Scheme 2). The selection of the substituents was based on docking scores and aimed to maintain binding interactions near the USP11 catalytic site.

**Scheme 2:**
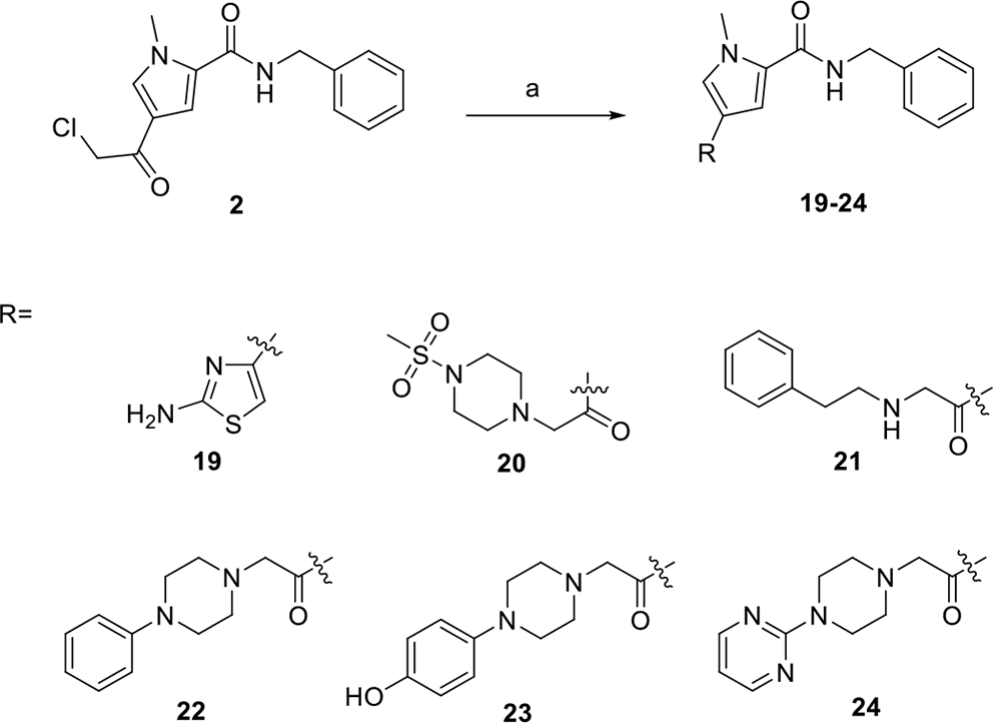
Synthesis of Compounds 19-24 as Derivatives from Compound 2[Fn sch2-fn2]

i Reagents and conditions: for compounds **19** (a) (i) compound **2** (1 equiv), thiourea (2 equiv), 1 mL of pyridine, MeOH, 4 h, rt. (ii) saturated NaHCO_3_ solution, flash column chromatography for purification, 30–50%. For compounds **20–24** (a) (i) compound **2** (1 equiv), K_2_CO_3_ (2 equiv), amine (1 equiv), MeCN, 16 h, reflux. (ii) cool, water, DCM (50 mL × 3), brine solution (50 mL), flash column chromatography for purification, 50–90%.

Using compounds **3–14** as starting point and analogous to the strategy adopted for compound **20**, a series of derivatives **25–35** was synthesized in which a methyl sulfonylpiperazine moiety was introduced in place of the chlorine atom on the original compounds (Scheme 3). To establish the relevance of the connectivity of the methyl sulfonylpiperazine group, compound **36** was synthesized.

**Scheme 3:**
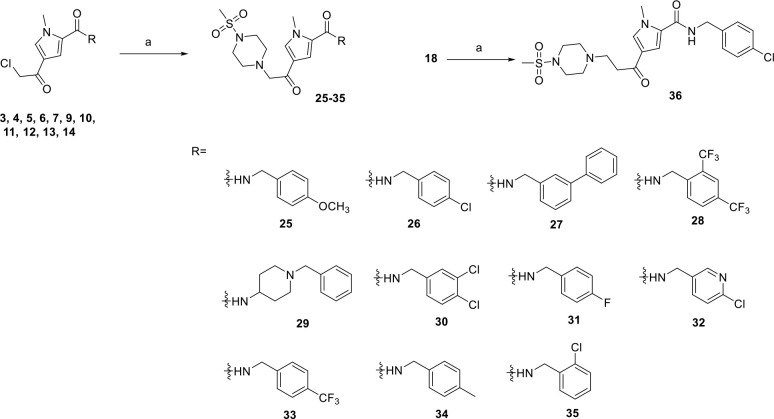
Synthesis of Methylsulfonyl Piperazine Derivatives[Fn sch3-fn3]

i Reagents and conditions: (a) (i) chloroacetyl derivatives (1 equiv), K_2_CO_3_ (2 equiv), 1-(methylsulfonyl)­piperazine (1 equiv), MeCN, 16 h, reflux. (ii) cool, water, DCM (50 mL × 3), brine solution (50 mL), flash column chromatography for purification, 70–90%.

Further modifications on the pyrrole nitrogen atom were carried out to explore the steric requirements at this position yielding compounds **38–40** (Scheme 4).

**Scheme 4:**
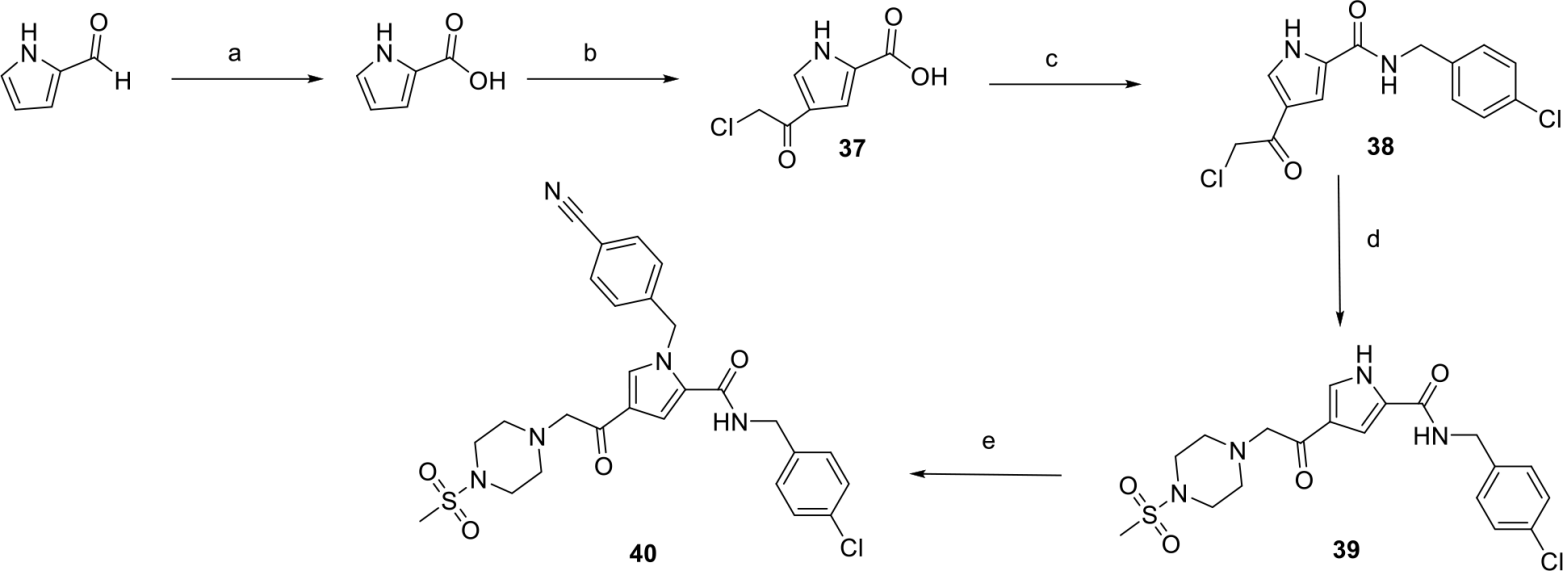
Synthesis of Unsubstituted Pyrrole Derivatives and *N*-Benzonitrile Derivative 39 and 40[Fn sch4-fn4]

i Reagents and conditions: (a) Pyrrol-2-carboxaldehyde (1 equiv), silver oxide (1.5 equiv), NaOH (2 equiv), methanol/water solvent, 2 h, 45%. (b) and (c) are the same for the synthesis of compounds **2–14**, 35 and 40%, respectively. (d) is the same for the synthesis of compounds **25–36**, 65% (e) (i) compound **39** (1 equiv), KOH (2 equiv), α-Bromo-p-tolunitrile (1.1 equiv), DMF, 50 °C, 16 h. (ii) Water, EtOAc, then DCM extract, flash column chromatography for purification, 15%.

The potency of the synthesized compounds was evaluated in a USP11 “IsoMim” enzyme activity assay,^41^ in which cleavage of a ubiquitin isopeptide bond mimetic was monitored using fluorescence polarization (Table 1). Moreover, to investigate the interaction of the compounds with USP11, changes in thermal stability of USP11 in the presence of selected inhibitors, in addition to inactive control compounds, were measured (Table 1).

**Table 1:** IC_50_ Values against USP11 Using the IsoMim FP Assay and the Shift in Melting Temperature Δ*T*_m_ for Compounds **2–40**

**Cpd**	**USP11 IC**_**50**_ **(μM)** ^a^	**USP11 Δ*T***_**m**_ **(°C)** ^a^	**Cpd**	**USP11 IC**_**50**_ **(μM)** ^a^	**USP11 Δ*T***_**m**_ **(°C)** ^a^
2	10.3 ± 0.14	–6.14 ± 0.40	**22**	>500	n.d.
3	12.5 ± 3.21	–7.05 ± 0.17	**23**	>500	n.d.
4	12.7 ± 2.08	–6.30 ± 0.89	**24**	>500	n.d.
5	9.18 ± 0.11	–5.98 ± 0.23	**25**	21.4 ± 2.22	–3.8 ± 0.12
6	12.5 ± 1.25	–5.17 ± 0.81	**26**	10.9 ± 0.83	–4.93 ± 0.14
7	2.59 ± 0.12	–7.02 ± 0.25	**27**	>500	n.d.
8	11.2 ± 0.30	–5.66 ± 0.30	**28**	>500	n.d.
9	9.24 ± 1.56	n.d.^b^	**29**	69.3 ± 7.23	n.d.
10	3.44 ± 0.17	–4.30 ± 0.14	**30**	102 ± 11.8	n.d.
11	10.5 ± 2.28	n.d.	**31**	202 ± 4.34	n.d.
12	11.7 ± 0.33	n.d.	**32**	12.2 ± 3.16	–2.08 ± 0.04
13	10.21 ± 0.13	n.d.	**33**	>500	n.d.
14	9.48 ± 2.40	n.d.	**34**	>500	n.d.
16^c^	>500	-0.09 ± 0.06	**35**	>500	n.d.
18	>500	n.d.	**36** ^c^	>500	–0.75 ± 0.16
19	>500	n.d.	**38**	21.5 ± 0.31	n.d.
20	48.0 ± 5.08	–2.17 ± 0.33	**39**	23.6 ± 2.26	–0.65 ± 0.08
21	186 ± 2.20	n.d.	**40**	33.1 ± 3.17	n.d.

a Data represent the mean ± SD of three independent experiments (*n* = 3).

b n.d. means not determined.

c Compounds **16** and **36** were tested in the DSF assay as inactive analogues.

While most of the chloroacetyl-containing compounds **3**–**14** were equipotent to the reported compound **2**, compounds **7** and **10** showed increased potency (Table 1). Compound **7** displayed the most pronounced inhibitory activity against USP11 with an IC_50_ of 2.59 μM (Table 1 and Figure 3). Additionally, removal of the chlorine atom in compound **16** resulted in complete loss of inhibitory activity, confirming the importance of this moiety.^36^ Furthermore, replacement of this group led to a significant drop in the inhibitory activity as shown for the synthesized compounds **19–24** (Table 1) with most compounds losing any measurable activity. Nevertheless, the methyl sulfonylpiperazine derivative **20** retained USP11 inhibitory capacity with IC_50_ = 48 μM (Table 1 and Figure S1).

**Figure 3:**
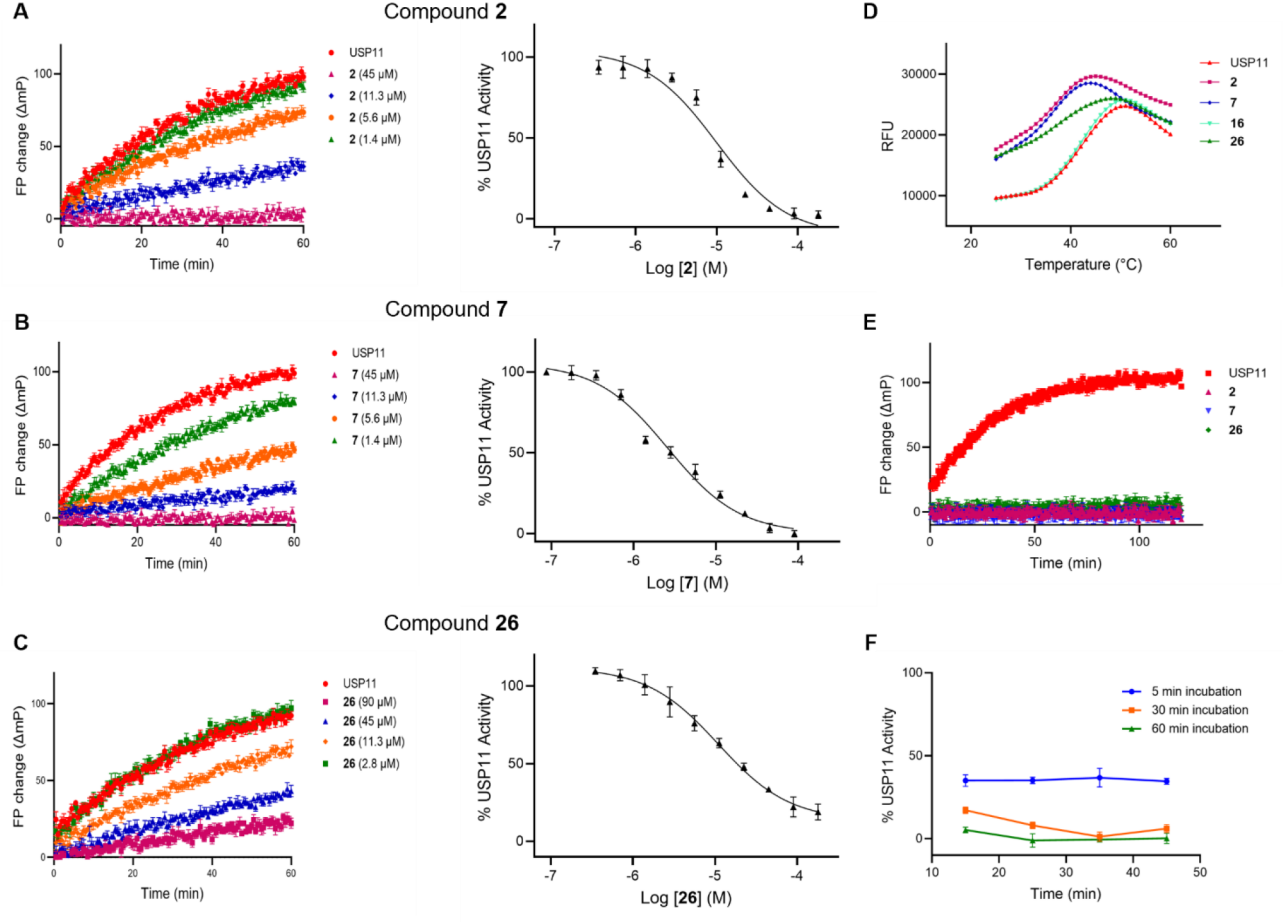
A: Left: Progression curve for USP11 enzyme activity measured by change in fluorescence polarization for compound **2** at 45–1.4 μM over 1 h, (mean ± SEM, *n* = 3); Right: Dose response curve for inhibition of USP11 enzyme activity by compound **2** measured as % of activity in the absence of compound **2**; the data points represent the mean ± SD of three independent experiments (*n* = 3) B: as under A for compound **7** C: as under A for compound **26** D: DSF assay of USP11 with compounds **2**, **7**, and **26** in addition to **16** as negative control, showing the melting curves where the relative fluorescence unit (RFU) values are plotted on the *Y*-axis and the temperature in °C on the *X*-axis. A leftward shift in *T*_m_ in the presence of **2**, **7**, and **26** was observed; the data points represent the mean of three independent experiments (*n* = 3). E: Enzyme jump dilution assay, showing the covalent nature for compounds **2**, **7**, **26**, the data points represent the mean ± SEM (*n* = 3). F: Activity of **7** against USP11 at 12.5 μM concentration measured as % of activity in the absence of **7** at three different incubation times 5, 30, and 60 min (mean ± SD, *n* = 3).

Substitution of the chlorine atom with methylsulfonyl piperazine (**25**-**36**) was associated with a decrease in the inhibitory activity against USP11 in most derivatives, with the exception of compounds **26** and **32**, which were equipotent to their chloroacetyl counterparts (Table 1).

Within the class of compounds around the methylsulfonyl piperazine scaffold, compound **26** (a 4-Cl derivative of **20**) exhibited the most potent inhibitory activity, showing 4-fold increase in potency compared to the nonsubstituted compound **20**. Moreover, replacing the phenyl ring of **26** with a pyridine ring slightly reduced the potency as observed for compound **32**. Compound **25** (the 4-methoxy derivative of **20**) was twice as potent as compound **20**. In contrast, the other derivatives revealed a significant decrease in activity compared to compound **20** (Table 1).

Increasing the carbon chain length between the carbonyl group and piperazine ring from one to two carbons (**36)**, resulted in significant decrease in USP11 inhibition, while the results showed that *N*-methylation of the pyrrole nitrogen atom is preferred, however, decrease in activity was observed with the unsubstituted pyrrole nitrogen and the benzonitrile substituent **39** and **40,** respectively (Table 1).

In the DSF assay, it can be observed that compounds **7** and **26** displayed a significant leftward shift in the USP11 melting temperature Δ*T*_m_ of −7.02 °C and −4.93 °C, respectively in comparison with **2**, which showed a −6.14 °C shift. For negative control compounds **16** and **36** a minor shift of the *T*_m_ of less than −1 °C was observed (Table 1, Figure 3D, and Figure S2A).

Compound **2** was reported as an irreversible covalent inhibitor.^36^ The binding nature of inhibitors **2**, **7**, and **26** was confirmed using an enzyme jump dilution assay, which showed results consistent with irreversible inhibition for **2** and **7**. Interestingly, compound **26** exhibited an unexpected slow off rate from USP11 (Figure 3E). In a similar manner, **7** exhibited increased USP11 inhibition with prolonged incubation time with 60% inhibition of the activity after 5 min of incubation and complete inhibition after 1 h incubation (Figure 3F).

Structure–activity relationship (SAR) for the tested compounds **19**-**40** revealed the requirement for the methyl sulfonyl piperazinyl moiety. The substitution on the pyrrole nitrogen to a methyl as in compound **26** is more favorable compared to the unsubstituted nitrogen of the ring or the introduction of an aromatic bulky substituent as the activity decreased in compounds **39** and **40**. Moreover, the one carbon spacer between the carbonyl ketone and the piperazine ring is important as extending the spacer led to a drastic drop in the activity (compound **36**). Finally, the preferred substituent on the amide nitrogen atom is a para- substituted phenyl ring as in compound **26** with the chlorine atom substituent being the most potent found to date (Figure 4).

**Figure 4:**
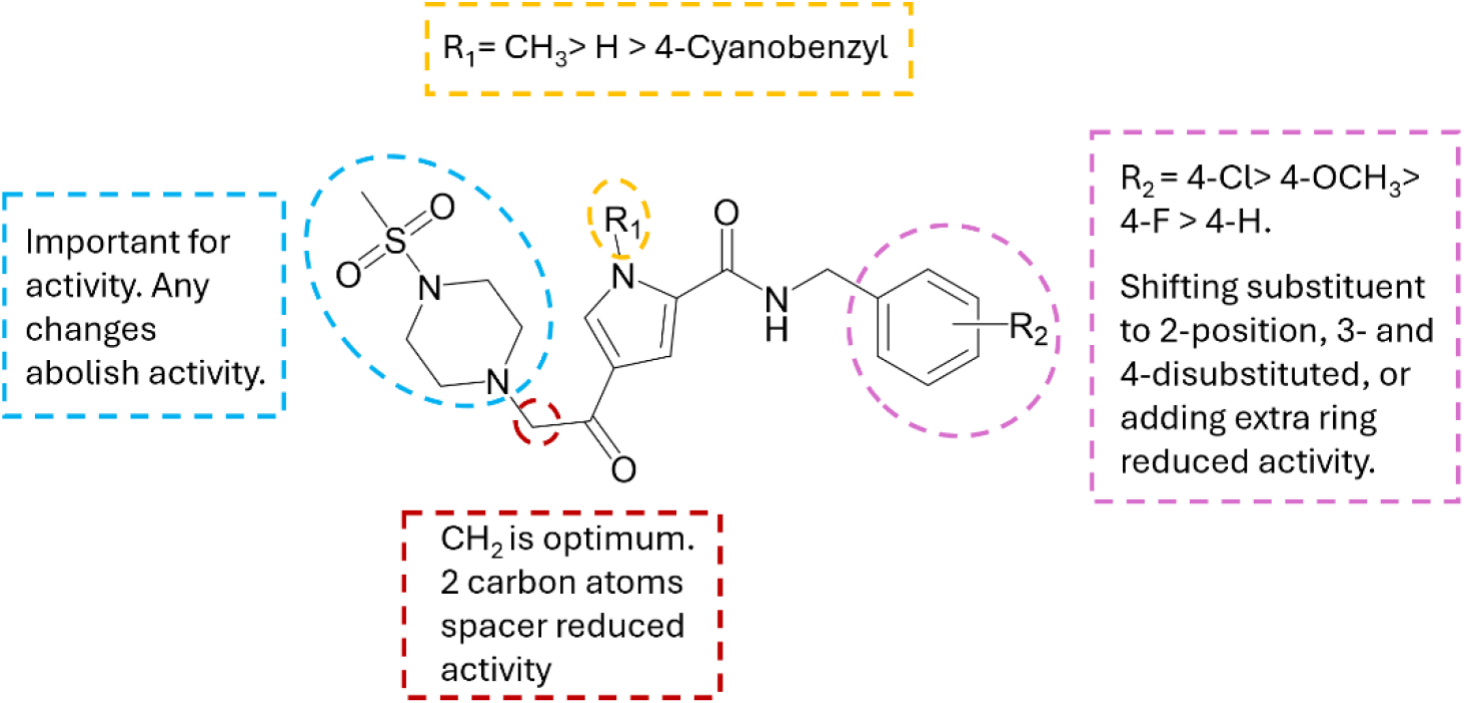
Structure Activity Relationships (SARs) for the synthesized compounds **19**-**40**.

### Potential Binding Mode of USP11 Inhibitors

Docking studies of compounds **7** and **26** indicated that both molecules can fit into the USP11 active site when Cys318 was converted to a glycine in the crystal structure of USP11 catalytic domain (PDB code: 8OYP). Docking poses positioned the compounds adjacent to the catalytic triad, forming direct hydrogen bonding interactions (Figure 5). Reintroduction of the cysteine led to steric clashes, as expected for covalent compounds involving the catalytic cysteine. The compounds are predicted to form significant interactions with key amino acid residues in proximity to the catalytic triad (Figure 5). Notably, both compounds exhibited a binding affinity score of −8.1 kcal/mol, indicating strong and favorable interactions within the active site. Compound **7** is predicted to form hydrogen-bond interactions with residues in the USP11 CCL region (Asn313, Cys318, Phe319), SL region (His399) and BL2 region (Gly887), while compound **26** also engages in hydrogen-bond interactions with the side chains of Gln398 and Asp400 in the SL region (Figures 1 and 5 and Supp. Figure S3).

**Figure 5:**
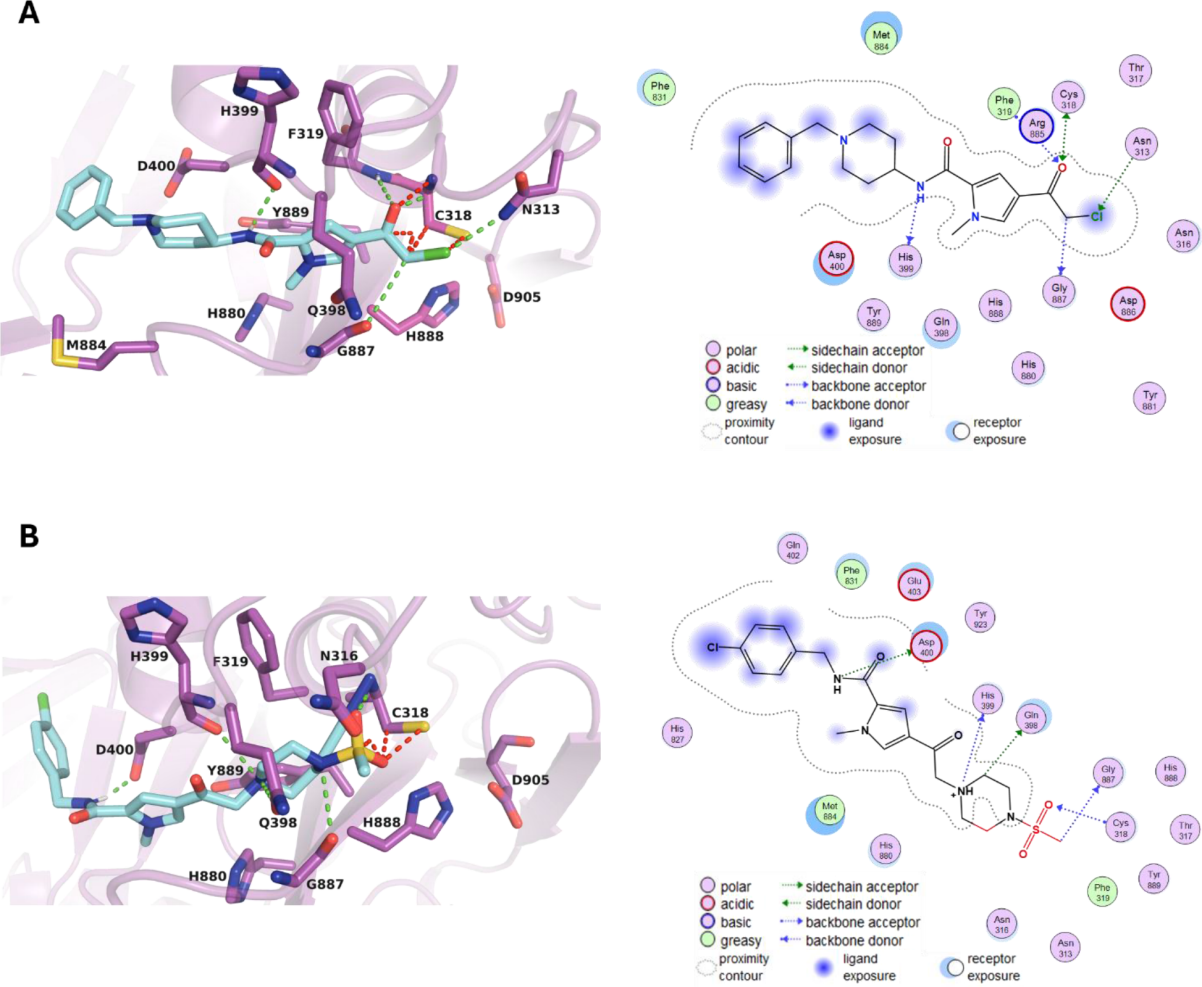
A: left: Prediction of binding mode of **7** in pale cyan in the USP11 active site in magenta showing the catalytic triad residues C318, H888, and D905, hydrogen bonding interactions are depicted in dashed green and clashes in dashed red lines, respectively; right: 2D representation of the protein-compound interaction. B: left: Binding of **26** in pale cyan at the USP11 catalytic site in magenta showing the catalytic triad C318, H888, and D905, hydrogen bonds interactions in dashed green and clashes in dashed red; right: 2D representation of the protein-compound interaction.

To evaluate the selectivity of the synthesized compounds for USP11, inhibition of USP11 was compared to inhibition of its two paralogues USP4 and USP15 using the IsoMim assay (Table 2). Analogous to previous observations, compound **2** preferentially inhibited USP4 over USP11 and USP15 (Figure S4). Compounds **7**, **20** and **25** did not inhibit USP15 and minimal inhibition was observed for compound **26** (Figure 6). The compounds showed differing degrees of selectivity for USP11 over USP4; compounds **20** and **25** displayed 4-fold selectivity for USP11 while compound **7** showed the highest USP11 selectivity of the compounds tested, with a 32-fold higher inhibitory potency for USP11 compared to USP4 (Table 2 and Figure 6). The results from the thermofluor assay are consistent with the IsoMim assay (Table 2 and Supp. Figure S2).

**Table 2:** Activity of Compounds 2, 7, and 26 against USP11, USP4, and USP15 and Selectivity Ratio Compared to USP11

	**IC**_**50**_ **(μM)** ^a^			Δ ***T***_**m**_ **(°C)** ^a^		
**Compound**	USP11	USP4	USP15	USP11	USP4	USP15
2	10.3 ± 0.14	2.0 ± 0.10 (0.19-fold)	27.3 ± 1.33 (2.6-fold)	–6.14 ± 0.40	–-6.67 ± 0.35	–3.79 ± 0.25
7	2.6 ± 0.12	82.7 ± 4.21 (31.8-fold)	>500 (>100-fold)	–7.02 ± 0.25	–3.07 ± 0.21	–1.82 ± 0.18
26	10.9 ± 0.83	18.1 ± 2.19 (1.7-fold)	>500 (>100-fold)	-4.93 ± 0.14	-4.69 ± 0.90	–0.03 ± 0.11

a Data represent the mean ± SD of three independent experiments (*n* = 3).

**Figure 6:**
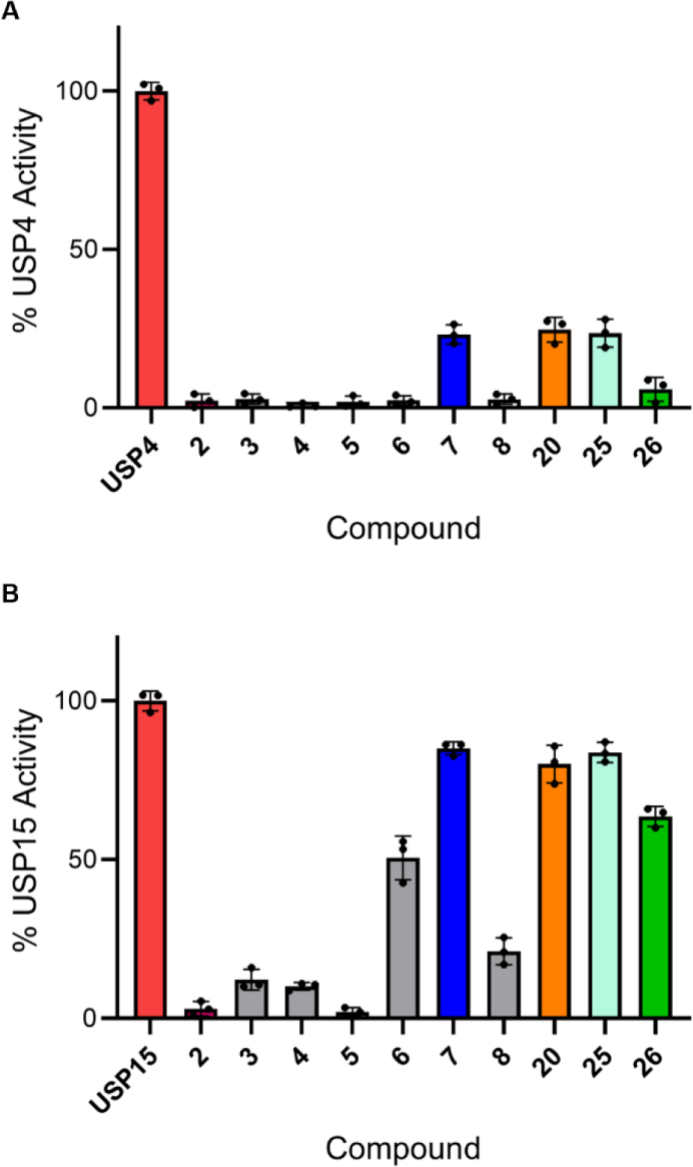
Inhibition of USP4 (A) and USP15 (B) by 45 μM of compound as indicated using the IsoMim assay. Data are expressed as % of enzyme activity in the absence of compound; the data points represent the mean ± SD of three independent experiments (*n* = 3).

### Evaluation of Cellular Activity of USP11 Inhibitors

USP11 has been implicated in carboplatin resistance of ovarian cancer cells, through stabilization of the protein BIP.^24^ Therefore, we evaluated whether compounds **7** and **26** (alongside compound **2**) could reduce the viability of platin-resistant ovarian cancer cells (PEO4). Compounds **2**, **7**, and **26** were tested across a range of concentrations (0.31, 0.63, 1.25, 2.5, 5, 7.5, and 10 μM) as inhibitors of PEO4 proliferation. Compounds **2** and **7** displayed significant inhibition of cell viability and appeared to be cytotoxic to the PEO4 ovarian cancer cells (Table 3; Figure 7A) since at higher concentrations complete inhibition was observed (Figure 7A). Compound **26** showed a more potent inhibition of cell viability (IC_50_ = 0.47 μM); however, since even at high concentration it did not affect the seeding levels of the cells, it appeared to be a cytostatic effect on the ovarian cancer cells, confirming its different mode of action.

**Table 3:** Activity of Compounds 2, 7, and 26 against Different Cell Lines

				**(SI)** ^b^	
**Cpd**	**PEO4 IC**_**50**_ **(μM)** ^a^	**MDA-MB-231 IC**_**50**_ **(μM)** ^a^	**MRC-5 IC**_**50**_ **(μM)** ^a^	**PEO4**	**MDA-MB-231**
**2**	3.1 ± 0.12	0.55 ± 0.05	2.8 ± 0.50	0.9	5
**7**	2.0 ± 0.08	2.3 ± 0.09	5.5 ± 0.10	2.8	2.4
**26**	0.47 ± 0.04	6.5 ± 0.35	>10	**> 21**	1.5

a Data represent the mean ± SD of three independent experiments (*n* = 3).

b The selectivity index calculated as IC_50_ (MRC-5)/IC_50_ (cancer cells).

**Figure 7:**
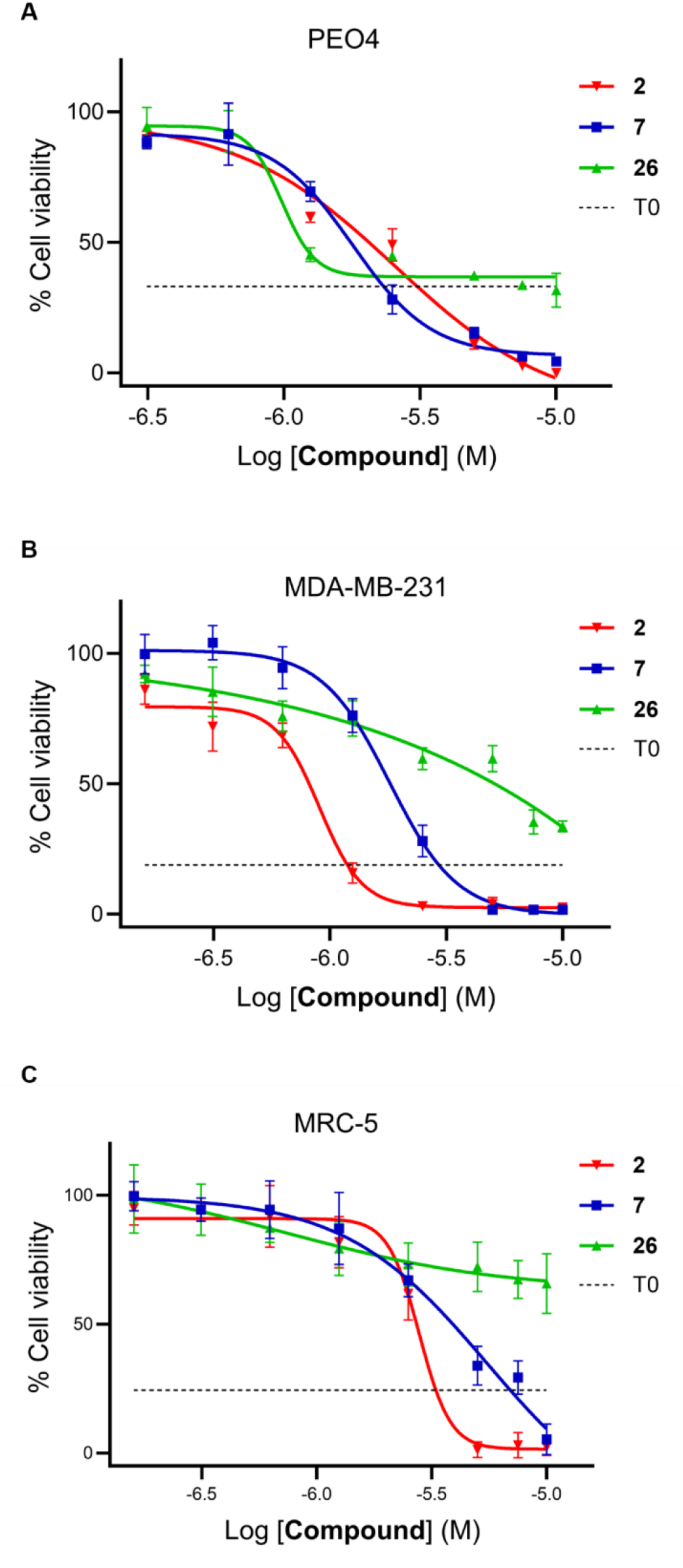
A: Ovarian cancer cells (PEO4) were seeded and treated with a concentration series of compounds **2**, **7**, and **26** (0.31, 0.63, 1.25, 2.5, 5, 7.5, and 10 μM) for 72 h. Relative cell viabilities were then determined using the MTS assay, with the viability of control cells set as 100%. B: Breast cancer cells (MDA-MB-231) were seeded and treated with a concentration series of compounds **2**, **7**, and **26** (0.16, 0.31, 0.63, 1.25, 2.5, 5, 7.5, and 10 μM) for 72 h. Relative cell viabilities were then determined using the MTS assay, with the viability of control cells set as 100%. C: Normal lung cells (MRC-5) were seeded and treated with a concentration series of compounds **2**, **7**, and **26** (0.16, 0.31, 0.63, 1.25, 2.5, 5, 7.5, and 10 μM) for 72 h. Relative cell viabilities were then determined using the MTS assay, with the viability of control cells set as 100%. The data points represent the mean ± SD of three independent experiments (*n* = 3).

USP11 has also been implicated in the proliferation of triple negative breast cancer cells.^42^ Evaluation of the antiproliferative activity of compound **2** on the MDA-MB-231 cell line (used as model for triple negative breast cancer) indicated that compound **2** exhibited significant cytotoxicity toward MDA-MB-231 cells at 1.25 μM, reducing overall cell viability to ∼16% and nearly completely inhibiting viability at higher concentrations. In contrast, compounds **7** and **26** showed weaker effects at the same concentration, maintaining ∼75% cell viability. Meanwhile, compound **26** was less cytotoxic at higher concentrations (Figure 7B).

To evaluate the cytotoxicity of **2**, **7**, and **26** against noncancerous cells, MRC-5 lung fibroblasts were used. Compound **2** exhibited the highest cytotoxicity, with a sharp decrease in cell viability at higher concentrations. Compound **7** also demonstrated cytotoxicity similar to **2**; however, it was slightly less cytotoxic. In contrast, **26** showed the lowest cytotoxic effect, maintaining viability above 50% at 10 μM, indicating a higher safety profile for this fibroblast cell line (Figure 7C). Furthermore, USP11-inactive compounds **16** and **36** (Table 1) were tested on the two cancer cell lines, PEO4 and MDA-MB-231, as negative controls. Compound **16** showed IC_50_ > 200 μM for both cell lines, while compound **36** displayed IC_50_ values of 55 and 46 μM, respectively (Supp. Figure S5).

## Discussion and Conclusion

The development of selective inhibitors targeting USP11 is crucial for investigating USP11 function and advancing therapeutic strategies in cancer and other diseases. In this study, a series of covalent inhibitors was synthesized and tested for their inhibitory effects on USP11, aiming to generate USP11 targeted inhibitors based on the previously reported covalent probe **2** that displayed some selectivity toward the USP11 paralogue USP4.^36^

The design of covalent inhibitors, **3–14**, was based on **2**. Evaluation of these compounds revealed that they were generally equipotent to **2**, with IC_50_ values in the low micromolar range. Notably, introducing a phenylpiperidine moiety instead of the phenyl ring, **7**, demonstrated the most potent inhibitory activity with an IC_50_ of 2.59 μM, indicating enhanced potency compared to **2** (IC_50_ = 10.27 μM). The chloroacetyl warhead is required for the inhibitory activity of these compounds, which was confirmed by the synthesis of compound **16,** which was inactive and contains an acetyl moiety in place of the chloroacetyl group.

In line with this observation, developing a new class of inhibitors lacking the irreversible chloroacetyl moiety by replacing it with several moieties (compounds **19–24)** led to a marked reduction in inhibitory activity. Most of these compounds displayed no USP11 inhibitory activity. However, **20**, a chloromethyl to sulfonylpiperazine replacement, exhibited a notable inhibitory effect with an IC_50_ of 48.04 μM, rendering it a reversible inhibitor candidate starting compound for further optimization.

The structural optimization of **20** led to the synthesis of a series of methylsulfonyl piperazine derivatives **25–35**. Among these, **26**, the addition of 4-chloro substituent on the phenyl ring of **20**, displayed a ∼4.5-fold increase in potency. On the other hand, the introduction of a 4-methoxy group in **25**, showed a ∼2.5-fold increase in potency. The addition of bulky substituents instead of the phenyl group, biphenyl and phenylpiperidine for **27** and **29** respectively, displayed reduced activity. Moreover, introducing 3,4-dichloro, 4-F, 4-methyl, 4-trifluoromethyl and/or changing the chlorine position from para- to ortho- abolished the activity as seen for **31**, **33–36**. This exploration and enhancement in potency for **26** suggests that the introduction of the methylsulfonyl piperazine scaffold is a promising strategy for improving the efficacy of USP11 inhibitors.

Further trials for increasing the carbon chain length between the carbonyl and the piperazine ring into two carbon atoms as in **36** led to a drastic reduction in activity. Furthermore, the optimized substituent on the pyrrole nitrogen is a methyl group as the unsubstituted **39** is 2-fold less active while introducing a bulky substituent on the nitrogen atom of **40** displayed a 3-fold reduction in activity.

Additionally, selectivity is a critical factor in the development of useful tool compounds and those for most therapeutic applications. The selectivity of the synthesized compounds was evaluated against USP11’s paralogues, USP4 and USP15. Compounds **7**, **20**, **25**, and **26** were selective for USP11, with significantly lower activity against USP4 and less or no activity observed against USP15. One reason for the consistently lower activity against USP15 may be that USP15 adopts an inactive conformation in the absence of substrate, whereby the catalytic triad cysteine is located 10 Å away from the catalytic histidine.^43^ Such conformations have so far not been observed for USP11 or USP4.^9,44^ Differences in the loop regions surrounding the active site between the paralogues have previously been shown to play a role in substrate interactions.^9^ It is likely that differences in these regions also influence inhibitor recognition (Figure S3). Further studies, such as crystals structures in complex with the compounds will be required to discern the molecular basis of the selectivity. The observed selectivity profile is desirable as it reduces the likelihood of off-target effects.^45,46^

The thermal stability of USP11 in the presence of various inhibitors was assessed using differential scanning fluorimetry (DSF), which measures shifts in the protein’s melting temperature (Δ*T*_m_). It has been reported that covalent compounds cause destabilization of the targeted proteins.^47^ For example, testing a library of covalent compounds targeting USP7 results in destabilization of the enzyme, leading to a downward shift in its melting temperature *T*_m_.^48^ Similarly, the tested compounds **7** and **26** displayed destabilization of USP11 with a significant decrease in its *T*_m_ similar to **2**, which is consistent with the FP assay results and supports the predicted binding of chloroacetyl-containing compounds **2** and **7** as well as the covalent nature of compound **26**. In contrast, negative control compounds such as **16** and **36** exhibited minimal shifts in USP11 *T*_m_, indicating negligible interaction with USP11. These results support effective interactions of the active compounds with USP11.

Furthermore, an enzyme jump dilution assay confirmed the covalent nature of inhibition of compounds **2** and **7**, and a slow off rate for **26**. While the methyl sulfonyl piperazine in **26** is not a classical covalent warhead, it may still contribute to covalent-like interactions with nucleophilic residues, which is supported by observations from the jump dilution assays that indicate sustained activity consistent with covalent binding. To support this, aryl ketones were reported as a reversible electrophilic warhead toward the nucleophilic cysteine and serine residues.^49^ Additionally, sulfonyl fluoride and vinyl sulfonamide groups have been reported to form stable adducts with tyrosine, lysine, and histidine residues which may enhance the compound’s selectivity and its slow off rate activity.^50,51^

The results of the MTS cell proliferation assays provide valuable insights into the differential effects of compounds **2**, **7**, and **26** on ovarian and breast cancer cell line viability with **26** particularly effective at lower concentrations. Additionally, **26** has less effect on the proliferation of a noncancerous fibroblast cell line, which suggests a more favorable safety profile and potential therapeutic window.

The higher activity of the tested compounds after 72 h incubation may be attributed to increased cellular sensitivity to their covalent nature,^52,53^ particularly for the irreversible compounds **2** and **7**. Also, compound **26** may exhibit covalent behavior, as suggested by results from the jump dilution assay. This highlights the potential of irreversible inhibitors to give sustainable inhibition even after compound washout,^54^ which would be a beneficial characteristic for therapeutic applications.^55^ Taken together, the results highlight **26** as promising candidate for further investigation.

Docking studies with compounds **7** and **26** indicated that both molecules are positioned close to the enzyme’s catalytic site. The analysis indicates a significant likelihood that these compounds can bind to the catalytic cysteine residue, a key mode of action for inhibiting cysteine proteases.^56^

Finally, the study identified and optimized the selectivity for USP11: **7** and **26** displayed improved selectivity and at least similar efficacy compared to previous compounds.^33–36^

The introduction of the methylsulfonyl piperazine group provided another slow off rate moiety for enhancing inhibitory activity, while jump dilution assays confirmed the covalent/slow off rate binding nature of these new inhibitors. These findings offer a promising direction for developing selective agents targeting USP11 to further probe USP11 function and potentially therapeutic applications.

## Experimental Section

### General Procedures, Chemistry

No unexpected or unusually high safety hazards were encountered. Chemicals and solvents were provided by commercial suppliers and were used without further purification. All reactions were monitored by TLC using Merck Silica Gel 60 Å F254 TLC plate and the visualization was under UV light (254 and 366 nm), or by LC–MS. All compounds were dried under a high vacuum. LC–MS data was collected on a Shimadzu UFLCXR HPLC system coupled to an Applied Biosystems API 2000 LC/MS/MS electrospray ionization (ESI). The column used was a Phenomenex Gemini-NX 3 μm-110Å C18, 50 × 2 mm at 40 °C. As eluent, a mixture of MeCN and H_2_O was used, containing 0.1% formic acid, the flow rate was 0.5 mL/min, and the UV detection was at 220 and 254 nm. Flash column chromatography was performed using silica gel 60, 230–400 mesh particle size (Sigma-Aldrich). Automated flash column normal phase chromatography was performed on a Biotage Isolera One system (ISO-1SV) equipped with a UV detector (200–400 nm) using silica high-performance (50 μm) cartridges. Methods were developed and run using Biotage Isolera (version: 3.3.0) software. NMR spectroscopy was performed using a Bruker AV­(III) HD 400 NMR spectrometer equipped with a 5 mm BBFO+ probe, recording ^1^H and ^13^C NMR at 400.25 and 100.66 MHz, respectively. NMR data was processed using MestReNova (version 15.0.0-34764) referencing spectra to residual solvents. Chemical shifts are quoted as δ: values in ppm; coupling constants *J* = are given in Hz, and multiplicities are described as follows: s, singlet; d, doublet; t, triplet; q, quartet; m, multiplet; dd, doublet of doublets; ddd, double doublet of doublets; dt, doublet of triplets; ddt, doublet of doublets of triplets; and bs, broad singlet. All compounds submitted for *in vitro* evaluation had a purity >95% determined by LC-MS. High-resolution mass spectrometry (HRMS) was achieved using a Bruker microTOF II MS with electrospray ionization (TOF ESI+).

### General Chemistry Procedure 1 for Synthesis of Intermediate Compounds 17 and 37

1-Methyl-1H-pyrrole-2-carboxylic acid (16.0 mmol, 1 equiv) in 30 mL DCM was dissolved. Then AlCl_3_ (32.0 mmol, 2 equiv) was added to the reaction mixture and stirred at 0 °C for 30 min, before adding the corresponding acyl chloride slowly (17.6 mmol, 1.1 equiv) and stirred overnight at 45 °C. The resulting reaction mixture was basified using a saturated NaHCO_3_ solution and filtered through Celite. The aqueous layer was acidified using conc. HCl and the obtained solid products were collected by filtration and further dried under a vacuum. Finally, extraction using ethyl acetate and drying using MgSO_4_ then evaporated on the rotary evaporator with a yield range from 30 to 55%.

#### 4-(3-Chloropropanoyl)-1-methyl-1*H-*pyrrole-2-carboxylic Acid 17

(30% yield, > 95% purity). ^1^H NMR (400 MHz, DMSO-*d*_6_) δ 12.69 (s, 1H), 7.90 (d, *J* = 1.9 Hz, 1H), 7.19 (d, *J* = 1.9 Hz, 1H), 3.94–3.84 (m, 5H), 3.25 (t, *J* = 6.7 Hz, 2H). ^13^C NMR (101 MHz, DMSO-*d*_6_) δ 191.53, 162.14, 134.10, 124.95, 123.49, 116.91, 41.63, 37.54. LC-MS calc. for C_9_H_10_ClNO_3_ = 215.03 found 216.1 [M + H] ^+^.

#### 4-(2-Chloroacetyl)-1*H*-pyrrole-2-carboxylic acid 37

(55% yield, > 99% purity). ^1^H NMR (400 MHz, DMSO-*d*_6_) δ 12.75 (s, 1H), 12.52 (s, 1H), 7.79 (dt, *J* = 3.3, 1.5 Hz, 1H), 7.14 (q, *J* = 1.8 Hz, 1H), 4.86 (d, *J* = 1.2 Hz, 2H). ^13^C NMR (101 MHz, DMSO-*d*_6_) δ 186.80, 161.92, 128.85, 125.54, 123.25, 114.56, 47.51. LC-MS calc. for C_8_H_9_NO_3_ = 167.16 found 168.1 [M + H] +.

### General Chemistry Procedure 2 for Synthesis of Compounds 2–14, 18 and 38

To a stirred solution of carboxylic acid derivatives (2a-2c) (3.0 mmol, 1 equiv) in 45 mL THF, EDC. HCl (3.6 mmol, 1.2 equiv) and HOBt (3.3 mmol, 1.1 equiv) were added at rt. A solution of the corresponding amine (3.6 mmol, 1.2 equiv) in 5 mL THF was added dropwise at rt. Then the reaction mixture was stirred for 16 h at rt. The resulting mixture was quickly poured into a saturated solution of citric acid (150 mL) and extracted with ethyl acetate (100 mL). The organic phase was separated and washed with saturated sodium bicarbonate solution (100 mL). The organic layer was then separated, dried over Na_2_SO_4_, filtered, and concentrated under reduced pressure. The resulting crude material was purified by flash chromatography (1% methanol in DCM) with an 18–63% yield. Spectral data for compounds **2** and **7** (Figures S6 and S7).

#### *N*-Benzyl-4-(2-chloroacetyl)-1-methyl-1*H-*pyrrole-2-carboxamide 2

(40% yield, > 97% purity). ^1^H NMR (400 MHz, CDCl_3_) δ 7.43 (d, *J* = 1.8 Hz, 1H), 7.43–7.27 (m, 5H), 7.05 (d, *J* = 1.8 Hz, 1H), 6.38 (s, 1H), 4.59 (d, *J* = 5.7 Hz, 2H), 4.39 (s, 2H), 4.03 (s, 3H); ^13^C NMR (101 MHz, DMSO-*d*_6_) δ 186.24, 160.99, 140.10, 133.31, 128.75, 128.00, 127.68, 127.21, 120.33, 112.58, 47.08, 42.41, 37.56; HRMS (TOF ESI+) *m*/*z* calc. for C_15_H_15_ClN_2_O_2_ [M + H]^+^ = 291.0895, found = 291.0883.

#### *N*-(4-Methoxybenzyl)-4-(2-chloroacetyl)-1-methyl-1*H-*pyrrole-2-carboxamide 3

(37% yield, > 99% purity). ^1^H NMR (400 MHz, CDCl_3_) δ 7.43 (d, *J* = 1.8 Hz, 1H), 7.32–7.24 (m, 2H), 7.01 (d, *J* = 1.8 Hz, 1H), 6.95–6.87 (m, 2H), 6.24 (s, 1H), 4.52 (d, *J* = 5.6 Hz, 2H), 4.40 (s, 2H), 4.03 (s, 3H), 3.83 (s, 3H)); ^13^C NMR (101 MHz, DMSO-*d*_6_) δ 186.23, 160.87, 158.66, 133.25, 132.03, 129.06, 128.07, 120.30, 114.15, 112.50, 55.53, 47.07, 41.87, 37.54; HRMS (TOF ESI+) *m*/*z* calc. for C_16_H_17_ClN_2_O_3_ [M + H]^+^ = 321.1000, found = 321.0992.

#### 4-(2-Chloroacetyl)-*N-*(4-chlorobenzyl)-1-methyl-1*H-*pyrrole-2-carboxamide 4

(35% yield, > 98% purity). ^1^H NMR (400 MHz, CDCl_3_) δ 7.45 (d, *J* = 1.8 Hz, 1H), 7.38–7.28 (m, 4H), 7.05 (d, *J* = 1.8 Hz, 1H), 6.34 (s, 1H), 4.56 (d, *J* = 5.8 Hz, 2H), 4.40 (s, 2H), 4.03 (s, 3H); ^13^C NMR (101 MHz, DMSO-*d*_6_) δ 186.24, 161.02, 139.16, 133.38, 131.76, 129.56, 128.70, 127.86, 120.35, 112.65, 47.08, 41.80, 37.55; HRMS (TOF ESI+) *m*/*z* calc. for C_15_H_14_Cl_2_N_2_O_2_ [M + H]^+^ = 325.0505, found = 325.0491.

#### *N-*([1,1′-Biphenyl]-3-ylmethyl)-4-(2-chloroacetyl)-1-methyl-1*H-*pyrrole-2-carboxamide 5

(28% yield, > 98% purity). ^1^H NMR (400 MHz, DMSO-*d*_6_) δ 8.94–8.85 (m, 1H), 7.88 (d, *J* = 11.5 Hz, 1H), 7.70–7.51 (m, 3H), 7.55–7.42 (m, 1H), 7.48 (s, 4H), 7.42–7.28 (m, 2H), 4.78 (d, *J* = 12.4 Hz, 2H), 4.54–4.45 (m, 2H), 3.92 (d, *J* = 12.5 Hz, 3H); ^13^C NMR (101 MHz, DMSO-*d*_6_) δ 186.24, 161.03, 140.77, 140.70, 140.66, 133.34, 129.44, 127.99, 127.95, 127.14, 126.87, 126.25, 125.68, 120.35, 112.60, 47.08, 42.53, 37.57; HRMS (TOF ESI+) *m*/*z* calc. for C_21_H_19_ClN_2_O_2_ [M + H]^+^ = 367.1208, found = 367.1208.

#### *N-*(2,4-Bis­(trifluoromethyl)­benzyl)-4-(2-chloroacetyl)-1-methyl-1*H-*pyrrole-2-carboxamide 6

(63% yield, > 99% purity). ^1^H NMR (400 MHz, DMSO-*d*_6_) δ 8.99 (t, *J* = 6.1 Hz, 1H), 8.01 (s, 3H), 7.89 (d, *J* = 1.8 Hz, 1H), 7.37–7.32 (m, 1H), 4.78 (d, *J* = 1.0 Hz, 2H), 4.59 (d, *J* = 5.9 Hz, 2H), 3.88 (s, 3H); ^13^C NMR (101 MHz, DMSO-*d*_6_) δ 186.26, 161.25, 143.73, 133.57, 130.65 (q, *J* = 33 Hz), 128.57, 127.58, 125.20, 122.49, 121.13, 120.42, 112.83, 47.08, 41.84, 37.53; HRMS (TOF ESI+) *m*/*z* calc. for C_17_H_13_ClF_6_N_2_O_2_ [M + H]^+^ = 427.0643, found = 427.0629.

#### *N-*(1-Benzylpiperidin-4-yl)-4-(2-chloroacetyl)-1-methyl-1*H-*pyrrole-2-carboxamide 7

(18% yield, >96% purity). ^1^H NMR (400 MHz, DMSO-*d*_6_) δ 8.08 (d, *J* = 7.8 Hz, 1H), 7.83 (d, *J* = 1.9 Hz, 1H), 7.38–7.28 (m, 5H), 7.26 (d, *J* = 7.0 Hz, 1H), 4.76 (s, 2H), 3.87 (s, 3H), 3.69 (s, 1H), 3.47 (s, 2H), 2.81 (d, *J* = 11.0 Hz, 2H), 2.00 (s, 2H), 1.74 (d, *J* = 12.2 Hz, 2H), 1.61–1.47 (m, 2H); ^13^C NMR (101 MHz, DMSO-*d*_6_) δ 186.24, 160.32, 139.14, 133.12, 129.20, 128.65, 128.25, 127.33, 120.18, 112.49, 62.60, 52.71, 47.08, 46.79, 37.48, 31.99; HRMS (TOF ESI+) *m*/*z* calc. for C_20_H_24_ClN_3_O_2_ [M + H]^+^ = 374.1630, found = 374.1636.

#### *N-*(Benzo­[d]­[1,3]­dioxol-5-ylmethyl)-4-(2-chloroacetyl)-1-methyl-1*H-*pyrrole-2-carboxamide 8

(25% yield, > 95% purity). ^1^H NMR (400 MHz, DMSO-*d*_6_) δ 8.77 (t, *J* = 6.1 Hz, 1H), 7.86 (d, *J* = 1.8 Hz, 1H), 7.32 (d, *J* = 1.9 Hz, 1H), 6.90–6.82 (m, 2H), 6.78 (dd, *J* = 7.9, 1.7 Hz, 1H), 5.98 (s, 2H), 4.76 (s, 2H), 4.30 (d, *J* = 6.0 Hz, 2H), 3.90 (s, 3H); ^13^C NMR (101 MHz, DMSO-*d*_6_) δ 186.23, 160.89, 147.68, 146.50, 133.97, 133.29, 127.99, 120.93, 120.31, 112.56, 108.47, 108.41, 101.27, 47.08, 42.24, 37.55; HRMS (TOF ESI+) *m*/*z* calc. for C_16_H_15_ClN_2_O_4_ [M + H]^+^ = 335.0793, found = 335.0784.

#### 4-(2-Chloroacetyl)-*N-*(3,4-dichlorobenzyl)-1-methyl-1*H-*pyrrole-2-carboxamide 9

(32% yield, > 98% purity). ^1^H NMR (400 MHz, DMSO-*d*_6_) δ 8.89 (t, *J* = 6.1 Hz, 1H), 7.88 (d, *J* = 1.8 Hz, 1H), 7.60 (d, *J* = 8.3 Hz, 1H), 7.55 (d, *J* = 2.0 Hz, 1H), 7.37–7.27 (m, 2H), 4.77 (s, 2H), 4.39 (d, *J* = 6.0 Hz, 2H), 3.89 (s, 3H); ^13^C NMR (101 MHz, DMSO-*d*_6_) δ 186.24, 161.08, 141.41, 133.47, 131.31, 130.99, 129.75, 129.72, 128.11, 127.70, 120.38, 112.77, 47.08, 41.48, 37.57; LC-MS calc. for C_15_H_13_Cl_3_N_2_O_2_ = 359.63 found 360.7 [M + H]^+^. HRMS (TOF ESI+) *m*/*z* calc. for C_15_H_13_Cl_3_N_2_O_2_ [M + Na]^+^ = 380.9935, found = 380.9922.

#### 4-(2-Chloroacetyl)-*N-*(4-fluorobenzyl)-1-methyl-1*H-*pyrrole-2-carboxamide 10

(17% yield, > 97% purity). ^1^H NMR (400 MHz, DMSO-*d*_6_) δ 8.85 (t, *J* = 6.2 Hz, 1H), 7.86 (d, *J* = 1.8 Hz, 1H), 7.38–7.29 (m, 3H), 7.15 (t, *J* = 8.8 Hz, 2H), 4.76 (d, *J* = 1.1 Hz, 2H), 4.38 (d, *J* = 6.0 Hz, 2H), 3.90 (s, 3H); ^13^C NMR (101 MHz, DMSO-*d*_6_) δ 186.23, 160.98, 136.28 (d, *J* = 3 Hz), 133.34, 129.67 (d, *J* = 8 Hz), 127.92, 120.33, 115.47 (d, *J* = 21 Hz), 112.62, 47.07, 41.74, 37.55; HRMS (TOF ESI+) *m*/*z* calc. for C_15_H_14_ClFN_2_O_2_ [M + H]^+^ = 309.0801, found = 309.0793.

#### 4-(2-Chloroacetyl)-*N*-((6-chloropyridin-3-yl)­methyl)-1-methyl-1*H*-pyrrole-2-carboxamide 11

(30% yield, > 95% purity). ^1^H NMR (400 MHz, DMSO-*d*_6_) δ 8.91 (t, *J* = 6.0 Hz, 1H), 8.37 (d, *J* = 2.5 Hz, 1H), 7.87 (d, *J* = 1.8 Hz, 1H), 7.82–7.76 (m, 1H), 7.50 (s, 1H), 7.32 (d, *J* = 1.9 Hz, 1H), 4.75 (s, 2H), 4.41 (d, *J* = 5.9 Hz, 2H), 3.89 (s, 3H); ^13^C NMR (101 MHz, DMSO-*d*_6_) δ 186.24, 161.13, 149.44, 149.19, 139.46, 135.20, 133.45, 127.67, 124.50, 120.36, 112.81, 47.08, 37.54; HRMS (TOF ESI+) *m*/*z* calc. for C_14_H_13_Cl_2_N_3_O_2_ [M + H]^+^ = 326.0458, found = 326.0448.

#### 4-(2-Chloroacetyl)-1-methyl-*N*-(4-(trifluoromethyl)­benzyl)-1*H*-pyrrole-2-carboxamide 12

(22% yield, > 99% purity). ^1^H NMR (400 MHz, DMSO-*d*_6_) δ 8.95 (t, *J* = 6.1 Hz, 1H), 7.88 (d, *J* = 1.9 Hz, 1H), 7.70 (d, *J* = 8.0 Hz, 2H), 7.53 (d, *J* = 8.0 Hz, 2H), 7.36 (t, *J* = 1.4 Hz, 1H), 4.77 (d, *J* = 1.0 Hz, 2H), 4.49 (d, *J* = 5.9 Hz, 2H), 3.89 (s, 3H); ^13^C NMR (101 MHz, DMSO-*d*_6_) δ 186.24, 161.12, 145.02, 133.44, 128.32, 127.77, 125.66 (q, *J* = 4 Hz), 120.38, 112.75, 47.08, 42.13, 37.55; HRMS (TOF ESI+) *m*/*z* calc. for C_16_H_14_ClF_3_N_2_O_2_ [M + H]^+^ = 359.0769, found = 359.0764.

#### 4-(2-Chloroacetyl)-1-methyl-*N*-(4-methylbenzyl)-1*H*-pyrrole-2-carboxamide 13

(39% yield, > 99% purity). ^1^H NMR (400 MHz, DMSO-*d*_6_) δ 8.80 (t, *J* = 6.1 Hz, 1H), 7.86 (d, *J* = 1.8 Hz, 1H), 7.32 (t, *J* = 1.4 Hz, 1H), 7.22–7.10 (m, 4H), 4.76 (d, *J* = 1.0 Hz, 2H), 4.35 (d, *J* = 6.0 Hz, 2H), 3.90 (s, 3H), 2.28 (s, 3H); ^13^C NMR (101 MHz, DMSO-*d*_6_) δ 186.23, 160.92, 137.05, 136.24, 133.27, 129.28, 128.04, 127.69, 120.31, 112.53, 47.08, 42.16, 37.55, 21.14; HRMS (TOF ESI+) *m*/*z* calc. for C_16_H_17_ClN_2_O_2_ [M + H]^+^ = 305.1051, found = 305.1038.

#### 4-(2-Chloroacetyl)-*N*-(2-chlorobenzyl)-1-methyl-1*H*-pyrrole-2-carboxamide 14

(37% yield, > 98% purity). ^1^H NMR (400 MHz, DMSO-*d*_6_) δ 8.86 (t, *J* = 6.0 Hz, 1H), 7.89 (d, *J* = 1.7 Hz, 1H), 7.49–7.42 (m, 1H), 7.40–7.28 (m, 4H), 4.78 (d, *J* = 1.0 Hz, 2H), 4.48 (d, *J* = 5.8 Hz, 2H), 3.90 (s, 3H); ^13^C NMR (101 MHz, DMSO-*d*_6_) δ 186.26, 161.14, 136.85, 133.44, 132.39, 129.58, 129.15, 129.05, 127.76, 127.66, 120.38, 112.85, 47.09, 40.36, 37.56; HRMS (TOF ESI+) *m*/*z* calc. for C_15_H_14_Cl_2_N_2_O_2_ [M + H]^+^ = 325.0505, found = 325.0493.

#### *N-*(4-Chlorobenzyl)-4-(3-chloropropanoyl)-1-methyl-1*H-*pyrrole-2-carboxamide 18

(35% yield, > 95% purity). ^1^H NMR (400 MHz, DMSO-*d*_6_) δ 8.84 (t, *J* = 6.2 Hz, 1H), 7.84 (d, *J* = 1.8 Hz, 1H), 7.39 (dd, *J* = 8.5, 1.7 Hz, 2H), 7.32 (dt, *J* = 5.9, 1.7 Hz, 3H), 4.38 (d, *J* = 6.0 Hz, 2H), 3.92–3.76 (m, 7H), 3.22 (t, *J* = 5.9 Hz, 2H); ^13^C NMR (101 MHz, DMSO-*d*_6_) δ 191.42, 161.17, 139.22, 133.11, 131.74, 129.54, 128.70, 123.20, 112.40, 41.77, 41.63, 37.45; LC-MS calc. for C_16_H_16_Cl_2_N_2_O_2_ = 338.06 found 339.1 [M + H] ^+^.

#### 4-(2-Chloroacetyl)-*N*-(4-chlorobenzyl)-1*H*-pyrrole-2-carboxamide 38

(19% yield, > 95% purity). ^1^H NMR (400 MHz, DMSO-*d*_6_) δ 12.31 (s, 1H), 8.88 (t, *J* = 6.1 Hz, 1H), 7.78 (dd, *J* = 3.3, 1.5 Hz, 1H), 7.42–7.37 (m, 2H), 7.35–7.29 (m, 3H), 4.83 (s, 2H), 4.43 (d, *J* = 6.0 Hz, 2H); ^13^C NMR (101 MHz, DMSO-*d*_6_) δ 186.77, 160.49, 139.10, 131.79, 129.72, 129.55, 128.79, 128.72, 128.54, 127.99, 122.96, 110.07, 47.30, 41.84; HRMS (TOF ESI+) *m*/*z* calc. for C_14_H_12_Cl_2_N_2_O_2_ [M + H]^+^ = 311.0349, found = 311.0339.

### General Chemistry Procedure 3 for Synthesis of Final Compound 19

To a solution of *N*-benzyl-4-(2-chloroacetyl)-1-methyl-1*H*-pyrrole-2-carboxamide **3** (0.69 mmol, 1 equiv) in 30 mL of MeOH were added thiourea (1.38 mmol, 2 equiv) and 1 mL of pyridine, and the mixture was stirred at room temperature for 4 h. The mixture was then concentrated, and the precipitate was washed with saturated NaHCO_3_ solution, filtered, and the resulting crude material was purified by flash chromatography (1% methanol in DCM) with a 50% yield.

#### 4-(2-Aminothiazol-4-yl)-*N-*benzyl-1-methyl-1*H-*pyrrole-2-carboxamide 19

(26% yield, > 98% purity). ^1^H NMR (400 MHz, DMSO-*d*_6_) δ 8.62 (t, *J* = 6.1 Hz, 1H), 7.31 (s, 3H), 7.37–7.19 (m, 3H), 7.15 (d, *J* = 1.8 Hz, 1H), 7.09 (d, *J* = 1.9 Hz, 1H), 6.86 (s, 2H), 6.38 (s, 1H), 4.39 (d, *J* = 6.0 Hz, 2H), 3.84 (s, 3H); ^13^C NMR (101 MHz, DMSO-*d*_6_) δ 168.37, 161.64, 146.41, 140.55, 128.69, 127.60, 127.08, 126.13, 125.77, 119.15, 110.37, 97.31, 42.29, 36.75; HRMS (TOF ESI^+^) *m*/*z* calc. for C_16_H_16_N_4_OS [M + H]^+^ = 313.1118, found = 313.1124.

### General Chemistry Procedure 4 for Synthesis of Final Compounds 20–35 and 39

To a stirred solution of compounds (3–13), (0.5 mmol, 1 equiv) in 20 mL of acetonitrile, K_2_CO_3_, (1.0 mmol, 2 equiv) was added, after 5 min, (0.5 mmol, 1 equiv) of the corresponding amine was added. Then, the solution was brought to reflux conditions and left for 16 h. The solution was then allowed to cool, and 50 mL of water was added. The organic layer was then extracted with dichloromethane (50 mL × 3) and washed with brine solution (50 mL). The combined organic extracts were dried with anhydrous sodium sulfate, filtered, and the solvent was evaporated. A Flash Chromatography Column was performed with 1% DCM/Methanol to separate the desired product with a yield of 10–90%. Spectral data for compound **26** (Figure S8).

#### *N-*Benzyl-1-methyl-4-(2-(4-(methylsulfonyl)­piperazin-1-yl)­acetyl)-1*H-*pyrrole-2-carboxamide 20

(25% yield, > 95% purity). ^1^H NMR (400 MHz, DMSO-*d*_6_) δ 8.80 (t, *J* = 6.1 Hz, 1H), 7.84 (d, *J* = 1.8 Hz, 1H), 7.32 (dd, *J* = 6.7, 3.7 Hz, 6H), 7.24 (dd, *J* = 7.9, 5.8 Hz, 1H), 4.40 (d, *J* = 6.0 Hz, 2H), 3.89 (s, 3H), 3.60 (s, 2H), 3.20–3.10 (m, 4H), 2.88 (s, 3H), 2.59 (t, *J* = 4.8 Hz, 4H); ^13^C NMR (101 MHz, DMSO-*d*_6_) δ 191.74, 161.15, 140.20, 132.83, 128.74, 127.64, 127.37, 127.19, 122.14, 112.49, 64.09, 52.45, 45.86, 42.36, 37.43, 34.19; HRMS (TOF ESI^+^) *m*/*z* calc. for C_20_H_26_N_4_O_4_S [M + H]^+^ = 419.1748, found = 419.1759.

#### *N-*Benzyl-1-methyl-4-(phenethylglycyl)-1*H-*pyrrole-2-carboxamide 21

(10% yield, >95% purity). ^1^H NMR (400 MHz, DMSO-*d*_6_) δ 8.80 (t, *J* = 6.1 Hz, 1H), 7.78 (d, *J* = 1.8 Hz, 1H), 7.37–7.26 (m, 7H), 7.29–7.21 (m, 3H), 7.19 (dd, *J* = 12.5, 5.3 Hz, 2H), 4.40 (d, *J* = 6.0 Hz, 2H), 3.88 (s, 3H), 3.80 (s, 2H), 2.76 (dq, *J* = 12.2, 6.6 Hz, 4H); ^13^C NMR (101 MHz, DMSO-*d*_6_) δ 193.49, 161.17, 140.81, 140.19, 132.34, 129.08, 128.74, 128.70, 127.66, 127.18, 126.31, 121.69, 112.18, 55.84, 51.11, 42.38, 37.41, 36.34; HRMS (TOF ESI+) *m*/*z* calc. for C_23_H_25_N_3_O_2_ [M + H]^+^ = 376.2020, found = 376.2018.

#### *N-*Benzyl-1-methyl-4-(2-(4-phenylpiperazin-1-yl)­acetyl)-1*H-*pyrrole-2-carboxamide 22

(48% yield, > 95% purity). ^1^H NMR (400 MHz, DMSO-*d*_6_) δ 8.81 (t, *J* = 6.1 Hz, 1H), 7.88 (d, *J* = 1.8 Hz, 1H), 7.37–7.27 (m, 5H), 7.27–7.16 (m, 3H), 7.01–6.90 (m, 2H), 6.77 (t, *J* = 7.3 Hz, 1H), 4.40 (d, *J* = 6.1 Hz, 2H), 3.89 (s, 3H), 3.57 (s, 2H), 3.16 (t, *J* = 5.0 Hz, 4H), 2.63 (t, *J* = 5.0 Hz, 4H); ^13^C NMR (101 MHz, DMSO-*d*_6_) δ 192.00, 161.18, 151.52, 140.22, 132.92, 129.37, 128.74, 127.63, 127.33, 127.17, 122.30, 119.24, 115.85, 112.59, 64.90, 53.31, 48.62, 42.36, 37.41; HRMS (TOF ESI+) *m*/*z* calc. for C_25_H_28_N_4_O_2_ [M + H]^+^ = 417.2285, found = 417.2296.

#### *N-*Benzyl-4-(2-(4-(4-hydroxyphenyl)­piperazin-1-yl)­acetyl)-1-methyl-1*H-*pyrrole-2-carboxamide 23

(36% yield, > 95% purity). ^1^H NMR (400 MHz, DMSO-*d*_6_) δ 8.80 (d, *J* = 4.2 Hz, 1H), 7.38–7.19 (m, 3H), 6.85–6.74 (m, 1H), 6.70–6.60 (m, 1H), 4.40 (d, *J* = 6.0 Hz, 1H), 3.89 (s, 1H), 3.57–3.45 (m, 1H), 2.99 (d, *J* = 4.5 Hz, 1H), 2.99–2.86 (m, 1H), 2.61 (t, *J* = 4.9 Hz, 2H); ^13^C NMR (101 MHz, DMSO-*d*_6_) δ 192.05, 161.18, 151.33, 144.69, 140.22, 132.91, 128.73, 127.63, 127.31, 127.17, 122.31, 119.28, 118.21, 115.95, 115.89, 112.59, 64.98, 53.53, 50.40, 42.36, 37.41; HRMS (TOF ESI+) *m*/*z* calc. for C_25_H_28_N_4_O_3_ [M + H]^+^ = 433.2234, found = 433.2247.

#### *N-*Benzyl-1-methyl-4-(2-(4-(pyrimidin-2-yl)­piperazin-1-yl)­acetyl)-1*H-*pyrrole-2-carboxamide 24

(60% yield, >98% purity). ^1^H NMR (400 MHz, DMSO-*d*_6_) δ 8.81 (t, *J* = 6.0 Hz, 1H), 8.35 (dd, *J* = 4.7, 1.1 Hz, 2H), 7.88 (d, *J* = 1.8 Hz, 1H), 7.37–7.29 (m, 4H), 7.33–7.20 (m, 3H), 6.62 (td, *J* = 4.8, 1.2 Hz, 1H), 4.40 (d, *J* = 6.0 Hz, 2H), 3.89 (s, 3H), 3.76 (t, *J* = 4.9 Hz, 4H), 3.56 (s, 2H), 2.57–2.47 (m, 4H); ^13^C NMR (101 MHz, DMSO-*d*_6_) δ 192.00, 161.67, 161.18, 158.37, 140.22, 132.92, 128.73, 127.63, 127.33, 127.17, 122.26, 112.57, 110.53, 64.90, 53.08, 43.71, 42.36, 37.42; HRMS (TOF ESI+) *m*/*z* calc. for C_23_H_26_N_6_O_2_ [M + H]^+^ = 419.2190, found = 419.2206.

#### *N-*(4-Methoxybenzyl)-1-methyl-4-(2-(4-(methylsulfonyl)­piperazin-1-Yl)­acetyl)-1*H-*pyrrole-2-carboxamide 25

(65% yield, > 98% purity). ^1^H NMR (400 MHz, DMSO-*d*_6_) δ 8.73 (t, *J* = 6.1 Hz, 1H), 7.82 (d, *J* = 1.9 Hz, 1H), 7.29 (d, *J* = 1.6 Hz, 1H), 7.22 (d, *J* = 8.2 Hz, 2H), 6.89 (d, *J* = 8.3 Hz, 2H), 4.32 (d, *J* = 6.2 Hz, 2H), 3.89 (s, 3H), 3.73 (d, *J* = 1.0 Hz, 3H), 3.59 (s, 2H), 3.13 (t, *J* = 4.9 Hz, 4H), 2.94–2.86 (m, 3H), 2.59 (t, *J* = 4.9 Hz, 4H); ^13^C NMR (101 MHz, DMSO-*d*_6_) δ 191.74, 161.04, 158.64, 132.76, 132.13, 129.02, 127.45, 122.12, 114.13, 112.42, 64.08, 55.52, 52.44, 45.86, 41.83, 37.42, 34.17; HRMS (TOF ESI+) *m*/*z* calc. for C_21_H_28_N_4_O_5_S [M + H]^+^ = 449.1853, found = 449.1850.

#### *N-*(4-Chlorobenzyl)-1-methyl-4-(2-(4-(methylsulfonyl)­piperazin-1-Yl)­acetyl)-1*H-*pyrrole-2-carboxamide 26

(44% yield, > 97% purity). ^1^H NMR (400 MHz, DMSO-*d*_6_) δ 8.82 (t, *J* = 6.1 Hz, 1H), 7.84 (d, *J* = 1.9 Hz, 1H), 7.42–7.36 (m, 2H), 7.35–7.28 (m, 3H), 4.38 (d, *J* = 5.9 Hz, 2H), 3.88 (s, 3H), 3.60 (s, 2H), 3.13 (t, *J* = 4.9 Hz, 4H), 2.88 (s, 3H), 2.59 (t, *J* = 4.9 Hz, 4H); ^13^C NMR (101 MHz, DMSO-*d*_6_) δ 191.74, 161.18, 139.25, 132.91, 131.73, 129.53, 128.69, 127.23, 122.16, 112.57, 64.09, 52.44, 45.86, 41.76, 37.43, 34.19; HRMS (TOF ESI+) *m*/*z* calc. for C_20_H_25_ClN_4_O_4_S [M + H]^+^ = 453.1358, found = 453.1361.

#### *N-*([1,1’-Biphenyl]-3-ylmethyl)-1-methyl-4-(2-(4-(methylsulfonyl)­piperazin-1-yl)­acetyl)-1*H-*pyrrole-2-carboxamide 27

(47% yield, > 98% purity). ^1^H NMR (400 MHz, DMSO-*d*_6_) δ 8.84 (t, *J* = 6.0 Hz, 1H), 7.84 (d, *J* = 1.8 Hz, 1H), 7.65 (d, *J* = 1.4 Hz, 1H), 7.67–7.53 (m, 3H), 7.51–7.27 (m, 6H), 4.48 (d, *J* = 6.0 Hz, 2H), 3.89 (s, 3H), 3.60 (s, 2H), 3.13 (t, *J* = 4.8 Hz, 4H), 2.88 (s, 3H), 2.59 (t, *J* = 5.0 Hz, 4H); ^13^C NMR (101 MHz, DMSO-*d*_6_) δ 191.75, 161.19, 140.87, 140.68, 132.87, 129.44, 129.42, 127.95, 127.37, 127.14, 126.84, 126.22, 125.65, 122.16, 112.50, 64.09, 52.44, 45.86, 42.48, 37.44, 34.18; HRMS (TOF ESI+) *m*/*z* calc. for C_26_H_30_N_4_O_4_S [M + H]^+^ = 495.2061, found = 495.2055.

#### *N-*(2,4-Bis­(trifluoromethyl)­benzyl)-1-methyl-4-(2-(4-(methylsulfonyl)­piperazin-1-yl)­acetyl)-1*H-*pyrrole-2-carboxamide 28

(90% yield, > 98% purity). ^1^H NMR (400 MHz, DMSO-*d*_6_) δ 8.95 (t, *J* = 6.0 Hz, 1H), 8.01 (s, 3H), 7.87 (d, *J* = 1.9 Hz, 1H), 7.32 (d, *J* = 1.8 Hz, 1H), 4.58 (d, *J* = 5.9 Hz, 2H), 3.87 (s, 3H), 3.60 (s, 2H), 3.13 (t, *J* = 4.7 Hz, 4H), 2.89 (d, *J* = 1.3 Hz, 3H), 2.60 (d, *J* = 4.9 Hz, 4H). ^13^C NMR (101 MHz, DMSO-*d*_6_) δ 191.75, 161.40, 143.84, 133.14, 130.64 (q, *J* = 33 Hz), 128.55, 126.96, 125.21, 122.49, 122.22, 121.11, 112.74, 64.14, 52.45, 45.86, 41.81, 37.41, 34.18. HRMS (TOF ESI+) *m*/*z* calc. for C_22_H_24_F_6_N_4_O_4_S [M + H]^+^ = 555.1495, found = 555.1498.

#### *N-*(1-Benzylpiperidin-4-yl)-1-methyl-4-(2-(4-(methylsulfonyl)­piperazin-1-yl)­acetyl)-1*H-*pyrrole-2-carboxamide 29

(35% yield, > 96% purity). ^1^H NMR (400 MHz, DMSO-*d*_6_) δ 8.02 (d, *J* = 8.0 Hz, 1H), 7.80 (s, 1H), 7.36–7.18 (m, 6H), 3.86 (s, 3H), 3.68 (d, *J* = 8.9 Hz, 1H), 3.60 (s, 2H), 3.46 (s, 2H), 3.13 (t, *J* = 4.6 Hz, 4H), 2.89 (s, 3H), 2.81 (d, *J* = 11.3 Hz, 2H), 2.59 (t, *J* = 4.9 Hz, 3H), 2.00 (t, *J* = 11.5 Hz, 2H), 1.79–1.66 (m, 2H), 1.54 (q, *J* = 11.4 Hz, 2H); ^13^C NMR (101 MHz, DMSO-*d*_6_) δ 192.44, 161.21, 149.60, 140.24, 132.91, 131.26, 128.73, 128.21, 127.62, 127.35, 127.25, 127.17, 122.45, 112.64, 69.61, 65.53, 49.79, 42.35, 38.25, 37.42; HRMS (TOF ESI+) *m*/*z* calc. for C_25_H_35_N_5_O_4_S [M + H]^+^ = 502.2483, found = 502.2485.

#### *N-*(3,4-Dichlorobenzyl)-1-methyl-4-(2-(4-(methylsulfonyl)­piperazin-1-yl)­acetyl)-1*H-*pyrrole-2-carboxamide 30

(83% yield, > 97% purity). ^1^H NMR (400 MHz, DMSO-*d*_6_) δ 8.85 (t, *J* = 6.0 Hz, 1H), 7.85 (d, *J* = 1.9 Hz, 1H), 7.63–7.52 (m, 2H), 7.35–7.27 (m, 2H), 4.39 (d, *J* = 6.0 Hz, 2H), 3.88 (s, 3H), 3.60 (s, 2H), 3.13 (t, *J* = 4.9 Hz, 4H), 2.89 (s, 2H), 2.59 (t, *J* = 5.0 Hz, 4H); ^13^C NMR (101 MHz, DMSO-*d*_6_) δ 191.74, 161.23, 141.51, 133.02, 131.29, 130.98, 129.68, 128.08, 127.07, 122.19, 112.68, 64.11, 52.45, 45.86, 41.45, 37.44, 34.19; HRMS (TOF ESI+) *m*/*z* calc. for C_20_H_24_Cl_2_N_4_O_4_S [M + H]^+^ = 487.0968, found = 487.0954.

#### *N-*(4-Fluorobenzyl)-1-methyl-4-(2-(4-(methylsulfonyl)­piperazin-1-yl)­acetyl)-1*H-*pyrrole-2-carboxamide 31

(66% yield, > 95% purity). ^1^H NMR (400 MHz, DMSO-*d*_6_) δ 8.80 (d, *J* = 6.1 Hz, 1H), 7.84 (d, *J* = 1.8 Hz, 1H), 7.33 (d, *J* = 2.6 Hz, 2H), 7.16 (d, *J* = 2.8 Hz, 3H), 4.39 (d, *J* = 6.0 Hz, 2H), 3.99–3.85 (m, 2H), 3.89 (s, 3H), 3.60 (s, 2H), 3.13 (t, *J* = 4.9 Hz, 4H), 2.89 (s, 3H), 2.59 (t, *J* = 4.9 Hz, 4H); ^13^C NMR (101 MHz, DMSO-*d*_6_) δ 191.70, 161.18, 136.28 (d, *J* = 3 Hz), 133.34, 129.67 (d, *J* = 8 Hz), 127.92, 120.33, 115.47 (d, *J* = 21 Hz), 112.62, 64.07, 52.44, 45.84, 41.77, 37.43, 34.19; HRMS (TOF ESI+) *m*/*z* calc. for C_20_H_25_FN_4_O_4_S [M + H]^+^ = 437.1653, found = 437.1651.

#### *N*-((6-Chloropyridin-3-yl)­methyl)-1-methyl-4-(2-(4-(methylsulfonyl)­piperazin-1-yl)­acetyl)-1*H*-pyrrole-2-carboxamide 32

(64% yield, > 95% purity). ^1^H NMR (400 MHz, DMSO-*d*_6_) δ 8.85 (t, *J* = 6.0 Hz, 1H), 8.36 (d, *J* = 2.4 Hz, 1H), 7.84 (d, *J* = 1.8 Hz, 1H), 7.78 (dd, *J* = 8.5, 2.5 Hz, 1H), 7.49 (d, *J* = 8.2 Hz, 1H), 7.30 (d, *J* = 1.6 Hz, 1H), 4.41 (d, *J* = 5.9 Hz, 2H), 3.88 (s, 3H), 3.60 (s, 2H), 3.13 (t, *J* = 4.8 Hz, 4H), 2.89 (d, *J* = 1.1 Hz, 3H), 2.59 (t, *J* = 4.9 Hz, 4H); ^13^C NMR (101 MHz, DMSO-*d*_6_) δ 191.73, 161.29, 149.41, 149.16, 139.44, 135.29, 133.00, 127.05, 124.49, 122.18, 112.71, 64.10, 52.44, 45.86, 39.51, 37.41, 34.19; HRMS (TOF ESI+) *m*/*z* calc. for C_19_H_24_ClN_5_O_4_S [M + H]^+^ = 454.1310, found = 454.1305.

#### 1-Methyl-4-(2-(4-(methylsulfonyl)­piperazin-1-yl)­acetyl)-*N*-(4-(trifluoromethyl)­benzyl)-1*H*-pyrrole-2-carboxamide 33

(75% yield, > 98% purity). ^1^H NMR (400 MHz, DMSO-*d*_6_) δ 8.90 (t, *J* = 6.1 Hz, 1H), 7.85 (d, *J* = 1.8 Hz, 1H), 7.70 (d, *J* = 8.0 Hz, 2H), 7.52 (d, *J* = 7.9 Hz, 2H), 7.34 (t, *J* = 1.6 Hz, 1H), 4.48 (d, *J* = 6.0 Hz, 2H), 3.88 (d, *J* = 1.1 Hz, 3H), 3.60 (s, 2H), 3.13 (t, *J* = 4.7 Hz, 4H), 2.89 (d, *J* = 1.2 Hz, 3H), 2.59 (t, *J* = 4.9 Hz, 4H); ^13^C NMR (101 MHz, DMSO-*d*_6_) δ 191.74, 161.28, 145.12, 132.98, 128.29, 127.14, 125.66 (q, *J* = 4 Hz), 122.19, 112.65, 64.11, 52.45, 45.86, 42.10, 37.43, 34.19, 14.56; HRMS (TOF ESI+) *m*/*z* calc. for C_21_H_25_F_3_N_4_O_4_S [M + H]^+^ = 487.1621, found = 487.1625.

#### 1-Methyl-*N*-(4-methylbenzyl)-4-(2-(4-(methylsulfonyl)­piperazin-1-yl)­acetyl)-1*H*-pyrrole-2-carboxamide 34

(64% yield, > 98% purity). ^1^H NMR (400 MHz, DMSO-*d*_6_) δ 8.75 (t, *J* = 6.1 Hz, 1H), 7.83 (d, *J* = 1.7 Hz, 1H), 7.30 (d, *J* = 1.6 Hz, 1H), 7.22–7.10 (m, 4H), 4.35 (d, *J* = 6.0 Hz, 2H), 3.89 (d, *J* = 1.2 Hz, 3H), 3.59 (s, 2H), 3.13 (t, *J* = 4.8 Hz, 4H), 2.88 (d, *J* = 1.2 Hz, 3H), 2.59 (t, *J* = 4.8 Hz, 4H), 2.28 (s, 3H); ^13^C NMR (101 MHz, DMSO-*d*_6_) δ 191.74, 161.09, 137.15, 136.20, 132.79, 129.27, 127.65, 127.42, 122.13, 112.44, 70.26, 64.09, 52.45, 45.86, 42.11, 37.43, 34.18, 21.14; HRMS (TOF ESI+) *m*/*z* calc. for C_21_H_28_N_4_O_4_S [M + H]^+^ = 433.1904, found = 433.1895.

#### *N*-(2-Chlorobenzyl)-1-methyl-4-(2-(4-(methylsulfonyl)­piperazin-1-Yl)­acetyl)-1*H*-pyrrole-2-carboxamide 35

(72% yield, > 99% purity). ^1^H NMR (400 MHz, DMSO-*d*_6_) δ 8.81 (t, *J* = 5.9 Hz, 1H), 7.86 (t, *J* = 1.6 Hz, 1H), 7.49–7.40 (m, 1H), 7.34 (ddt, *J* = 14.5, 7.5, 1.8 Hz, 4H), 4.47 (d, *J* = 5.8 Hz, 2H), 3.89 (s, 3H), 3.61 (s, 2H), 3.14 (t, *J* = 4.8 Hz, 4H), 2.89 (d, *J* = 1.2 Hz, 3H), 2.60 (t, *J* = 4.9 Hz, 4H); ^13^C NMR (101 MHz, DMSO-*d*_6_) δ 191.76, 161.30, 136.93, 132.99, 132.36, 129.57, 129.09, 129.02, 127.66, 127.13, 122.19, 112.76, 64.13, 52.45, 45.87, 37.44, 34.19; HRMS (TOF ESI+) *m*/*z* calc. for C_20_H_25_ClN_4_O_4_S [M + H]^+^ = 453.1358, found = 453.1360.

#### *N-*(4-Chlorobenzyl)-1-methyl-4-(3-(4-(methylsulfonyl)­piperazin-1-Yl)­propanoyl)-1*H-*pyrrole-2-carboxamide 36

(19% yield, > 97% purity). ^1^H NMR (400 MHz, DMSO-*d*_6_) δ 8.81 (t, *J* = 6.1 Hz, 1H), 7.81 (d, *J* = 1.7 Hz, 1H), 7.43–7.35 (m, 2H), 7.35–7.27 (m, 3H), 4.38 (d, *J* = 6.1 Hz, 2H), 3.88 (d, *J* = 1.3 Hz, 3H), 3.08 (t, *J* = 4.9 Hz, 4H), 2.93–2.84 (m, 5H), 2.70 (t, *J* = 7.1 Hz, 2H), 2.49 (s, 4H); ^13^C NMR (101 MHz, DMSO-*d*_6_) δ 193.74, 161.23, 139.26, 132.85, 131.73, 129.54, 128.69, 127.40, 123.50, 112.45, 53.19, 52.26, 45.85, 41.76, 37.41, 36.72, 34.11; HRMS (TOF ESI+) *m*/*z* calc. for C_21_H_27_ClN_4_O_4_S [M + H]^+^ = 467.1514, found = 467.1522.

#### *N*-(4-Chlorobenzyl)-4-(2-(4-(methylsulfonyl)­piperazin-1-Yl)­acetyl)-1*H*-pyrrole-2-carboxamide 39

(20% yield, > 96% purity). ^1^H NMR (400 MHz, DMSO-*d*_6_) δ 12.15 (s, 1H), 8.83 (t, *J* = 6.0 Hz, 1H), 7.73 (dd, *J* = 3.2, 1.6 Hz, 1H), 7.39 (d, *J* = 8.2 Hz, 2H), 7.35–7.26 (m, 3H), 4.42 (d, *J* = 5.9 Hz, 2H), 3.62 (s, 2H), 3.12 (t, *J* = 4.8 Hz, 4H), 2.88 (s, 3H), 2.59 (t, *J* = 4.9 Hz, 4H); ^13^C NMR (101 MHz, DMSO-*d*_6_) δ 192.38, 160.64, 139.20, 131.77, 129.53, 128.71, 127.90, 127.28, 124.75, 110.04, 64.20, 52.40, 45.89, 41.81, 34.14; HRMS (TOF ESI+) *m*/*z* calc. for C_19_H_23_ClN_4_O_4_S [M + H]^+^ = 439.1201, found = 439.1199.

### General Chemistry Procedure 5 for the Synthesis of the Final Compound 40

To a solution of *N*-(4-chlorobenzyl)-4-(2-(4-(methylsulfonyl)­piperazin-1-yl)­acetyl)-1*H*-pyrrole-2-carboxamide **39** (0.12 mmol, 1.0 equiv) in DMF (5 mL), KOH (0.24 mmol, 2.0 equiv) was added, and the mixture was stirred for 10 min at room temperature. α-Bromo-p-tolunitrile (0.13 mmol, 1.1 equiv) was then added gradually. The reaction mixture was stirred at 50 °C for 16 h. After completion, the mixture was quenched with water and extracted with EtOAc and DCM. The combined organic extracts were dried over Na_2_SO_4_, filtered, and concentrated under reduced pressure. The residue was purified by flash column chromatography to give compound **40** in 15% yield.

#### *N*-(4-Chlorobenzyl)-1-(4-cyanobenzyl)-4-(2-(4-(methylsulfonyl)­piperazin-1-yl)­acetyl)-1*H*-pyrrole-2-carboxamide 40

(15% yield, >95% purity). ^1^H NMR (400 MHz, DMSO-*d*_6_) δ 8.86 (t, *J* = 6.1 Hz, 1H), 8.08 (d, *J* = 1.9 Hz, 1H), 7.80 (d, *J* = 8.1 Hz, 2H), 7.40–7.31 (m, 3H), 7.26 (d, *J* = 8.2 Hz, 2H), 7.18 (d, *J* = 8.2 Hz, 2H), 5.70 (s, 2H), 4.31 (d, *J* = 6.0 Hz, 2H), 3.65 (s, 2H), 3.13 (t, *J* = 4.8 Hz, 4H), 2.88 (s, 3H), 2.60 (t, *J* = 4.9 Hz, 4H); ^13^C NMR (101 MHz, DMSO-*d*_6_) δ 191.92, 162.78, 160.95, 144.59, 139.06, 132.91, 132.67, 131.72, 129.38, 128.61, 128.14, 126.65, 122.80, 119.16, 113.23, 110.55, 64.14, 52.40, 51.78, 45.87, 41.70, 36.26, 34.18; HRMS (TOF ESI+) *m*/*z* calc. for C_27_H_28_ClN_5_O_4_S [M + H]^+^ = 554.1623, found = 554.1608.

### Protein Expression and Purification

The USP11 catalytic domain was expressed in*Escherichia coli* BL21­(DE3)-RIL cells grown in 2xYT broth with 50 μg/mL Kanamycin and 35 μg/mL Chloramphenicol. Once the culture reached an OD600 of ∼ 0.6, overexpression was induced by adding 0.5 mM IPTG, and the cultures were grown for 16 h at 18 °C. Cells were harvested, resuspended in 50 mM Tris-Cl, pH 8, 300 mM NaCl, 20 mM imidazole, 10% glycerol and lysed through sonication. The lysate was loaded onto a HisTrap chelating column precharged with nickel ions, and the protein was eluted using an imidazole gradient. Fractions containing the protein were collected, concentrated, and subjected to further purification by size-exclusion chromatography using a Superdex 75 16/600 column with gel filtration buffer as previously described.^9^

### Preparation of Ub^L73P^-Ub-GGGC-Fluorescein-5-maleimide (DiUb^3G^-FM)

The preparation of the assay reagent DiUb^3G^-FM harboring a fluorescein moiety for the fluorescence polarization-based IsoMim assay was performed according to the method described previously and involved the expression and purification of a linear diubiquitin variant (Ub^L73P^-Ub-GGGC) followed by Fluorescein-5-maleimide labeling.^41^

### Fluorescence Polarization-Based IsoMim Deubiquitination Activity Assay

Reactions were performed in triplicate in small volume black 384 well plates (Greiner) in a final reaction volume of 50 μL. USP11, USP4 or USP15 was diluted in 0.01 M Phosphate-buffered saline (PBS). To each well, 15 μL of DMSO control (0.5% final concentration) or inhibitor was added in triplicate and was incubated with 15 μL of diluted USP (10 nM final concentration) or assay buffer (negative control) for 30 min at room temperature, as reported.^41^ Reactions were initiated by the addition of 15 μL of the prepared diubiquitin probe (10 nM final concentration) and read immediately and every 30 s for 1 h. Reading of the plates was performed using a PerkinElmer Envision 2104 Multilabel plate-reading spectrophotometer using 480 nm excitation and 535 nm emission filters, suitable for measurement of fluorescein. Fluorescence polarization was determined by measuring the parallel and perpendicular fluorescence emission intensity with respect to the polarized excitation light and is expressed in millipolarization (mP) units. In the dose response curve, data from three independent experiments were combined, normalized and presented as percentage values; data were fitted using nonlinear regression in GraphPad Prism with error bars representing the standard deviation SD.

### Differential Scanning Fluorimetry

Differential scanning fluorimetry (DSF) experiments were carried out in 96-well PCR plates and measured with a CFX96 Touch Real-Time PCR instrument (Bio-Rad). Protein unfolding was monitored by measuring the fluorescence of SYPRO Orange dye. The standard assay conditions were 50 mM HEPES pH 7.4 and 100 mM NaCl as buffer, with protein final concentrations of 5 μM and SYPRO Orange dye at 3x final concentration. All assays were performed in a final volume of 50 μL. The plates were sealed with a clear, optical foil (Bio-Rad) before starting the heating process in the PCR instrument. For all experiments, a heating rate of 1 °C/min was used, and the cycle was repeated 70x. The fluorescence was detected by using the FRET filter set (491 nm excitation peak, 586 nm emission peak). To determine the stabilization/destabilization of USP11, USP4, and USP15 different compounds were tested with a final compound concentration of 80 μM. Control wells containing the protein without compounds, compounds only and 1% DMSO were included in each experiment. Melting temperature shifts (Δ*T*_m_) were calculated for the tested compounds and the analysis of data was performed using Bio-Rad CFX Manager program, Excel, and GraphPad Prism. The *T*_m_ was determined by plotting the fluorescence values (RFU) or the first derivative -d­(RFU)/dT against temperature and fitting the data to the Boltzmann equation using GraphPad Prism with Δ*T*_m_ represented as mean ± SD from three independent experiments (n = 3).

### Enzyme Jump Dilution Assay

For measuring drug target residence times, preincubation of 1 μM USP11 (100-fold the working concentration) with inhibitors **2**, **7**, and **26** at 10 × IC_50_ concentration for 1 h at room temperature was performed. Subsequently, 100-fold dilution was carried out to adjust the compound concentration to 10 times lower than the respective IC_50_ value, i.e., 0.5 μL of USP11/Inhibitor solution was added to a 49.5 μL solution containing buffer and the probe in a 384-well plate. Well solutions were mixed and read immediately as well as every 30 s for 2 h using a PerkinElmer Envision 2104 Multilabel plate-reading spectrophotometer with 480 nm excitation and 535 nm emission filters. Data analysis was performed using GraphPad Prism.

### MTS Assay

To determine the cell viability and cytotoxicity of compounds **2**, **7**, and **26**, with compounds **16** and **36** as USP11-inactive controls, 2500 cells/well of ovarian cells (PEO4), breast cancer MDA-MB-231, and normal fibroblast MRC-5 were seeded in 96-well plates overnight. T0 was measured as the cell viability after 24 h. Then, cells were treated for 72 h with different concentrations of the compounds (0.08–10 μM) or DMSO as a control, which were incubated at 37 °C and 5% CO_2_ throughout the entire experiment. The cell viability was then evaluated by MTS assay. Twenty microliters of the MTS CellTiter 96 Cell Proliferation Assay reagent (Promega Corporation) were added to each well. After 2 h of incubation at 37 °C, the absorbance of the colored product was measured at wavelength 490 nm on a PerkinElmer Envision 2104 Multilabel plate-reader. For IC_50_ determination, the data were fitted using nonlinear regression in GraphPad Prism, with error bars representing the standard deviation SD.

### Docking

Docking studies were performed using the crystal structure of the USP11 catalytic domain (PDB ID: 8OYP^9^), in which the ubiquitin was removed, and the catalytic cysteine was *in silico* mutated to glycine. Compounds were drawn using ChemDraw 20.0 and docked using AutoDock Vina 1.1.2 ^57^ within UCSF Chimera version 1.17.^58^ The docking grid box was centered at x = −21.55 Å, y = −9.52 Å, z = 20.57 Å, with dimensions of 32.48 Å × 32.27 Å × 28.26 Å, selected to include the catalytic triad residues Cys318, His888, and Asp905 and the substrate binding site. The docking poses were visualized using the PyMOL Molecular Graphics System version 2.5.2 (Schrödinger, LLC),^59^ while detailed 2D ligand–residue interactions were generated with Molecular Operating Environment (MOE) 2020.09 (Chemical Computing Group ULC, 910–1010 Sherbrooke St. W., Montreal, QC H3A 2R7, 2025). These highlight hydrogen bonding and other interactions between the docked compounds and USP11 residues as used for other predictions.^60^

## Supplementary Material





## Data Availability

Data Availability Statement: The data underlying this study are available in the published article and its Supporting Information.

## Notes

Dose–response curve for **20** against USP11; compounds **2**, **7**, and **26** thermal shift curves against USP11, USP4, and USP15; sequence alignment of USP11, USP4, and USP15 catalytic core domains; dose–response curve for **2** against USP4 and USP15; activity of compounds **16** and **36** against PEO4 and MDA-MB-231 cells; and ^1^H NMR and ^13^C NMR spectra of compounds **2**, **7**, and **26** (PDF)
Compounds; SMILES; USP11 IC50 (μM); USP4 IC50 (μM); USP15 IC50 (μM); PEO4 IC50 (μM); MDA-MB-231 IC50 (μM); MRC-5 IC50 (μM) (CSV)

## Abbreviations

BRCA2: Breast cancer gene 2
DSF: Differential scanning fluorimetry
DUBs: Deubiquitinating enzymes
DUSP-UBL: Domain present in USP—Ubiquitin-like
ERα: Estrogen receptor α
EtOAc: Ethyl acetate
FM: Fluorescein-5-maleimide
FP: Fluorescence polarization
HEPES: 4-(2-Hydroxyethyl)-1-piperazineethanesulfonic acid
IPTG: Isopropyl β-D-1-thiogalactopyranoside
IsoMim: Isopeptide bond mimetic
MeCN: Acetonitrile
mP: Millipolarization
MTS: 3-(4,5-Dimethylthiazol-2-yl)-5-(3-carboxymethoxyphenyl)-2-(4-sulfophenyl)-2H-tetrazolium
RFU: Relative fluorescence units
TGFβ: Transforming growth factor-β
*T*_m_: melting temperature
Ub: ubiquitin
USP: Ubiquitin-specific protease
