# Supplementary material for: Design, Synthesis, and Evaluation of Selective Ubiquitin-Specific Protease 11 (USP11) Inhibitors
Source: ACS Omega. 2025 Dec 5;10(49):60555–71. doi: 10.1021/acsomega.5c08248 (PMC12713474; doi:10.1021/acsomega.5c08248)

Supporting Information

Design, Synthesis, and Evaluation of Selective Ubiquitin-Specific Protease 11 (USP11) Inhibitors

Mostafa A. Hassan [1, 2], Lodewijk V. Dekker [1], Michael J. Stocks [1], Ingrid Dreveny [1*]*

[1] Biodiscovery Institute, School of Pharmacy, University of Nottingham, Nottingham, NG7 2RD, United Kingdom.

[2] Department of Medicinal Chemistry, Faculty of Pharmacy, Assiut University, Assiut 71526, Egypt.

Email: michael.stocks@nottingham.ac.uk or ingrid.dreveny@nottingham.ac.uk

List of content

Figure S1. Activity of compound **20** against USP11 S2

Figure S2. DSF assay results for compounds **2**, **7**, **26** against USP11, USP4 and USP15 S3

Figure S3. Sequence alignment of USP11, USP4 and USP15 catalytic core domains S4

Figure S4. Dose response curve for **2** against USP4 and USP15 S5

Figure S5. Activity of compounds **16** and **36** against PEO4 and MDA-MB-231 cells S6

Figure S6. ^{1}H NMR and ^{13}C NMR for the key compound **2** S7

Figure S7. ^{1}H NMR and ^{13}C NMR for the key compound **7** S8

Figure S8. ^{1}H NMR and ^{13}C NMR for the key compound **26** S9

Figure S1. Activity of compound **20** against USP11

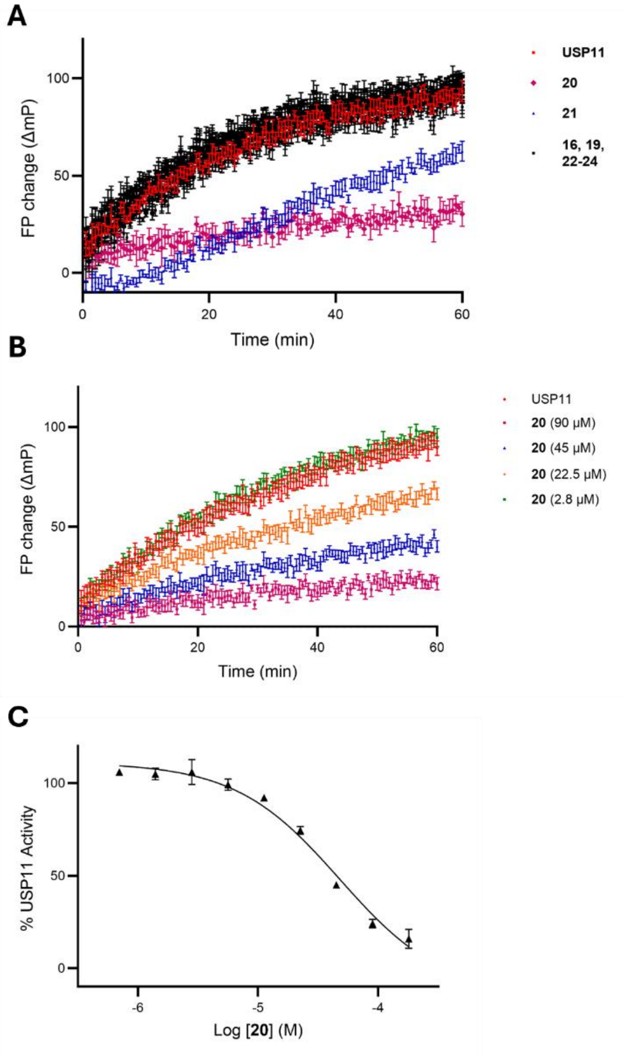

Figure S1. **A**: Progression curve for USP11 enzyme activity measured by change in fluorescence polarization for compounds **16, 19-24** at a concentration of 45 μM over 1 h; **B**: Progression curve for USP11 enzyme activity measured by change in fluorescence polarization for compound **20** at 90-2.8 μM over 1 h. (mean ± SEM, n = 3). **C:** Dose response curve for inhibition of USP11 enzyme activity by compound **20** measured as % of activity in the absence of compound **20**; the data points represent the mean ± SD of three independent experiments (n = 3).

Figure S2. DSF assay results for compounds **2**, **7**, **26** against USP11, USP4 and USP15

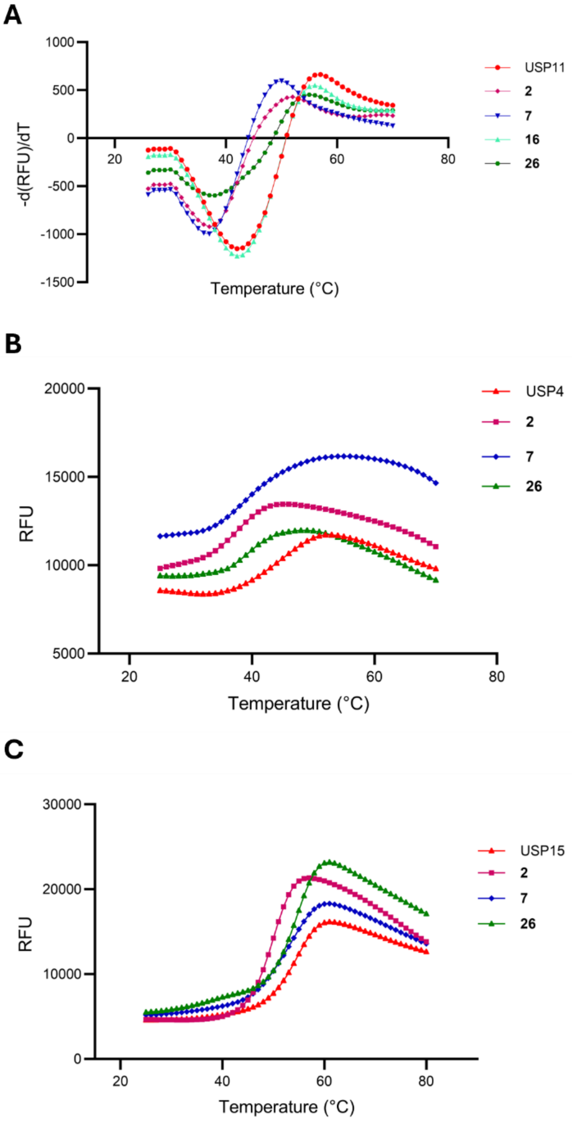

Figure S2. **A**: First derivative melt curves (−d(RFU)/dT vs. temperature) of USP11 with 1% DMSO alone (red dots) or with compound **16** (green triangles) as negative controls and compounds **2**, **7**, and **26** as indicated. A shift in the USP11 Tm in the presence of **2**, **7**, and **26** indicates destabilization (leftward shift). **B**: USP4 melting curves in the presence and absence of compounds **2**, **7**, and **26** where the relative fluorescence unit (RFU) values are shown on the Y-axis and temperature in °C on the X-axis. A leftward shift in Tm in the presence of **2**, **7**, and **26** indicates destabilization of the protein. **C**: DSF assay of USP15 in the presence and absence of compounds **2**, **7**, and **26** as indicated where the relative fluorescence unit (RFU) values are plotted on the Y-axis and temperature in °C on the X-axis. A leftward shift in the USP15 Tm in the presence of **2** was observed indicating destabilization of the protein; the data points represent the mean of three independent experiments (n = 3)**.**

Figure S3. Sequence alignment of USP11, USP4 and USP15 catalytic core domains

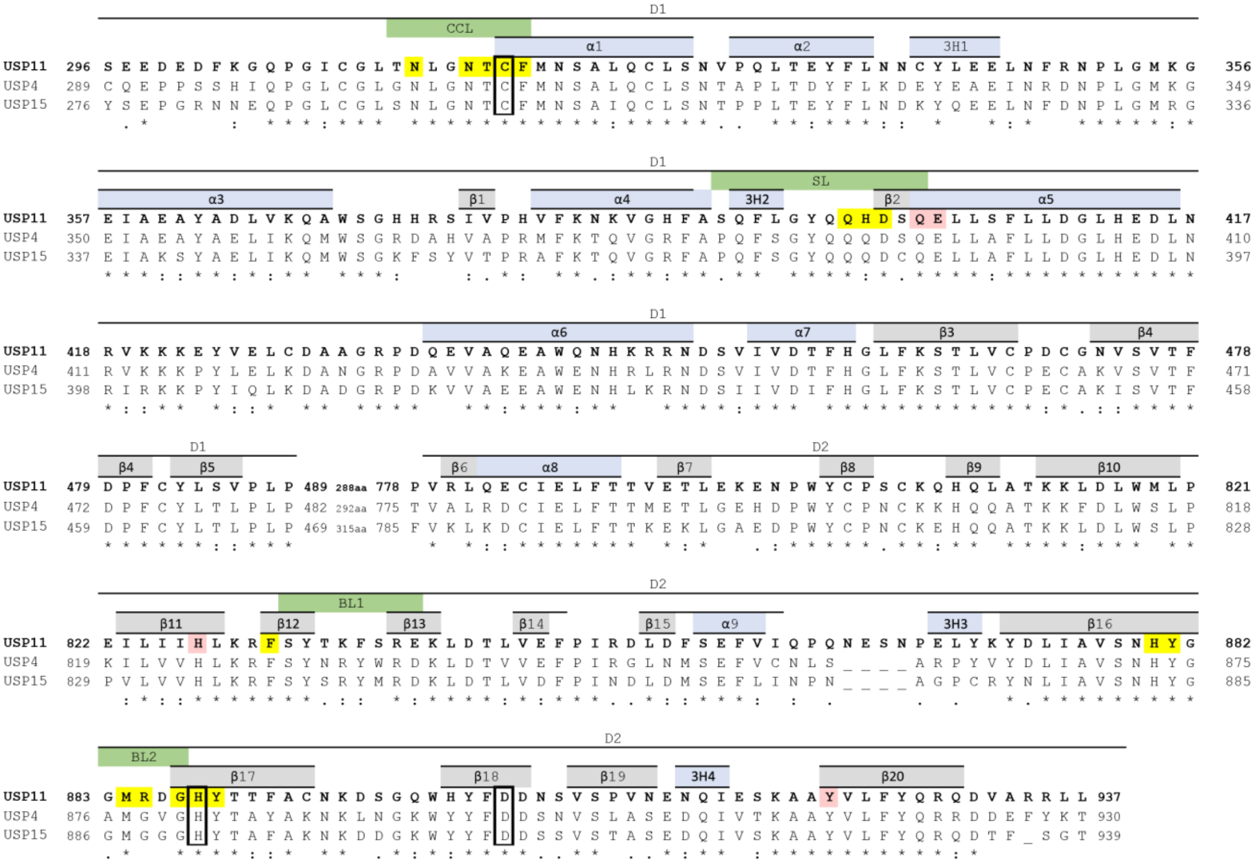

Figure S3. Sequence alignment of the catalytic core domains of *Hs* USP11 (UniProt: P51784), USP4 (UniProt: Q13107) and USP15 (UniProt: Q9Y4E8) indicating the two sub-domain halves (D1, D2) and secondary structure elements above the sequences. Flexible active site loop regions are shaded in green and labelled as CCL (catalytic cleft loop USP11 residues T^{312}-F^{319}), SL (switching loop, residues S^{391}-Q^{402}), BL1 (blocking loop 1, residues S^{832}-R^{838}) and BL2 (blocking loop 2, residues G^{882}-D^{886}). Catalytic triad residues are boxed and residues predicted to interact with compound **7** are shaded yellow, whereas additional residues likely involved in interacting with compound **26** are shaded in light orange.

Figure S4. Dose response curve for **2** against USP4 and USP15

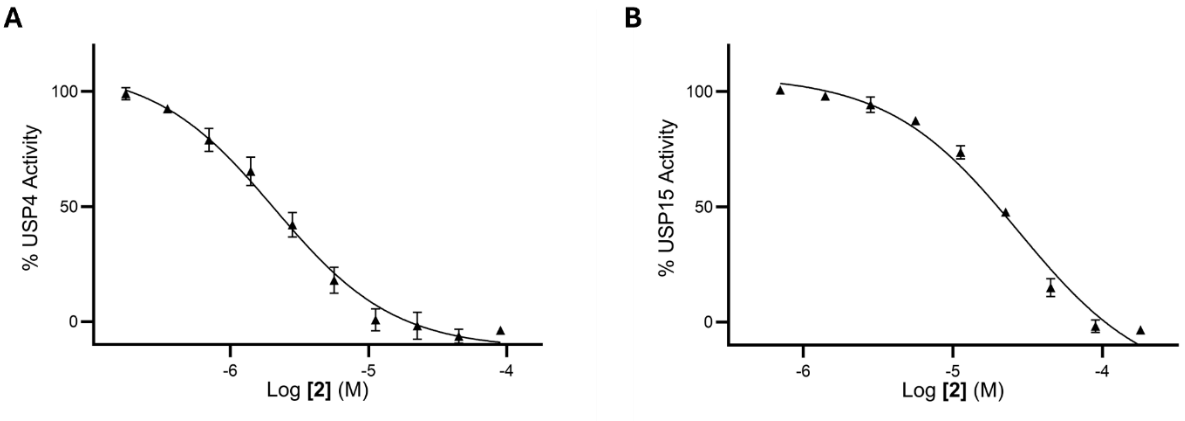

Figure S4. **A** and **B**: Dose response curves for compound **2** against USP4 and USP15 with FP change in ΔmP on the Y-axis and Logarithm concentration of compound **2** on the X-axis measured as % of activity in the absence of compound **2**; the data points represent the mean ± SD of three independent experiments (n = 3).

Figure S5. Activity of compounds **16** and **36** against PEO4 and MDA-MB-231 cells

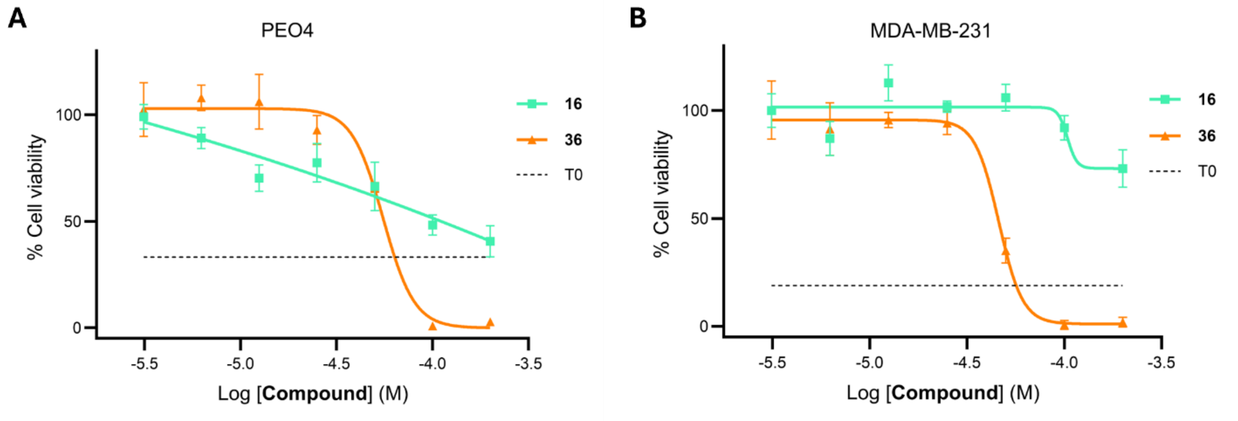

Figure S5. **A**: Ovarian cancer cells (PEO4) were seeded and treated with a concentration series of compounds **16** and **36** (3.13, 6.25, 12.5, 25, 50, 100, and 200 µM) for 72 h. Relative cell viabilities were then determined using the MTS assay, with the viability of control cells set as 100%. Error bars represent the standard deviation. **B**: Breast cancer cells (MDA-MB-231) were seeded and treated with a concentration series of compounds **16** and **36** (3.13, 6.25, 12.5, 25, 50, 100, and 200 µM) for 72 h. Relative cell viabilities were then determined using the MTS assay, with the viability of control cells set as 100%. Error bars represent the standard deviation SD (n = 3).

Figure S6. ^{1}H NMR and ^{13}C NMR for compound **2**

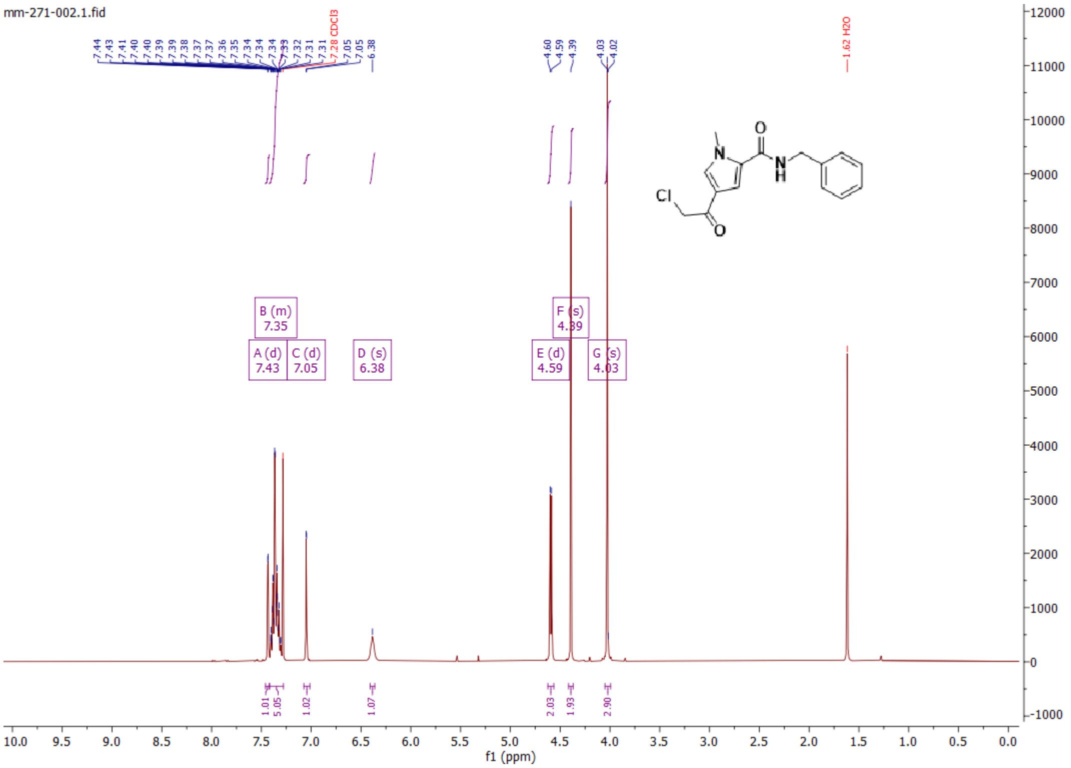

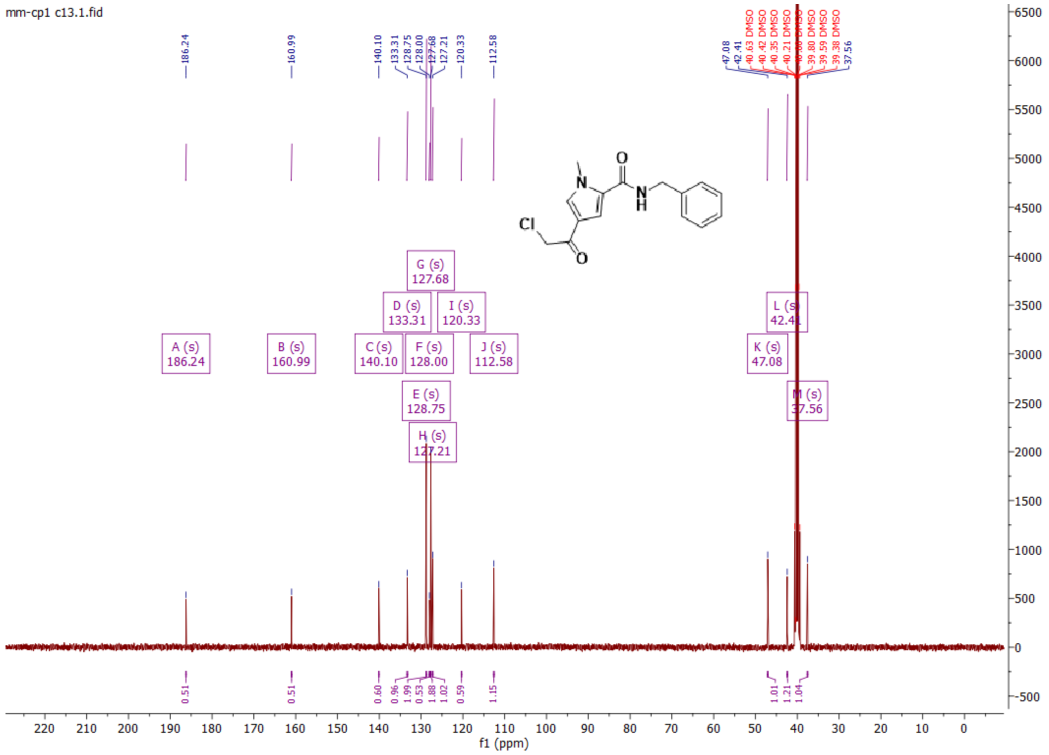

Figure S7. ^{1}H NMR and ^{13}C NMR for the key compound **7**

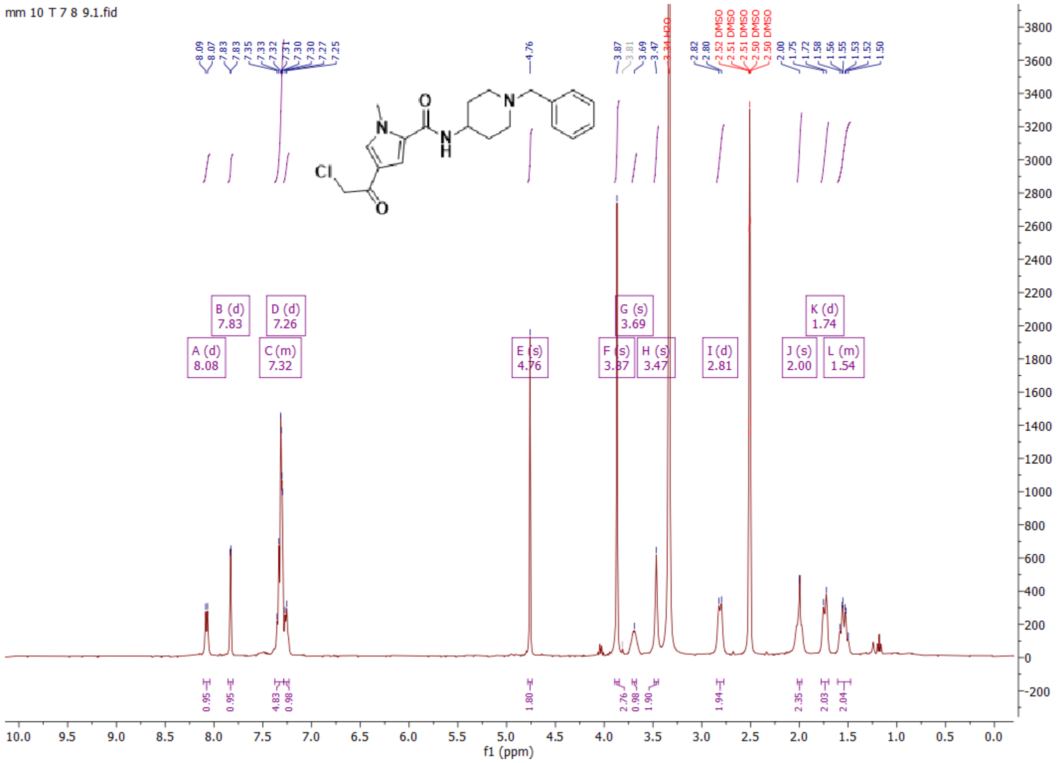

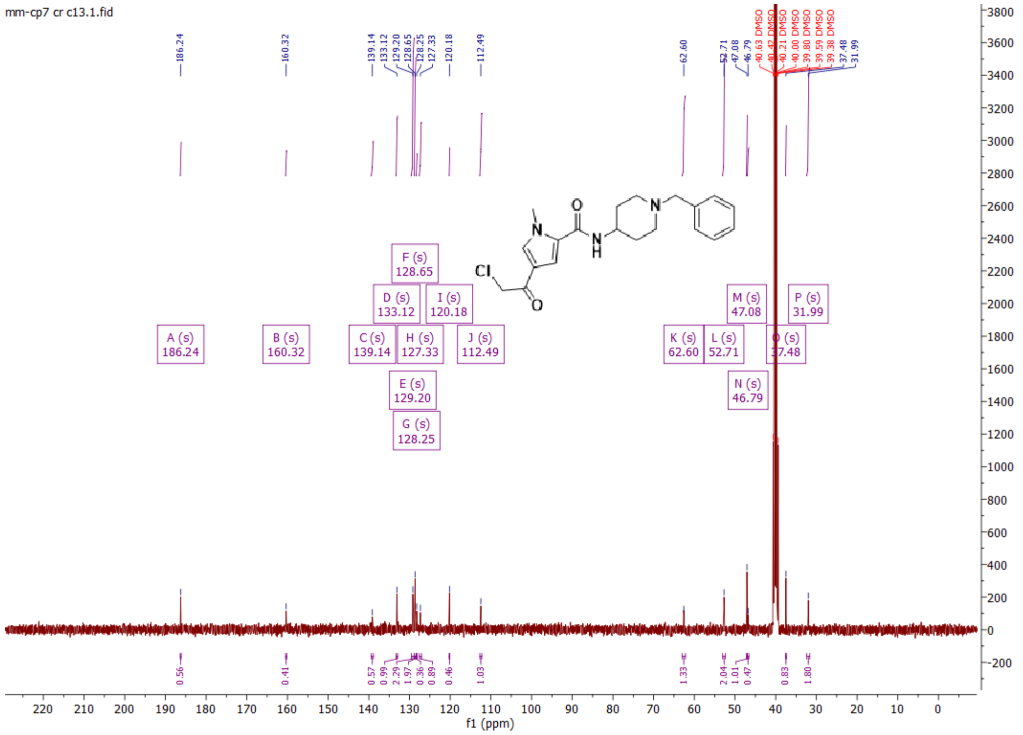

Figure S8. ^{1}H NMR and ^{13}C NMR for the key compound **26**

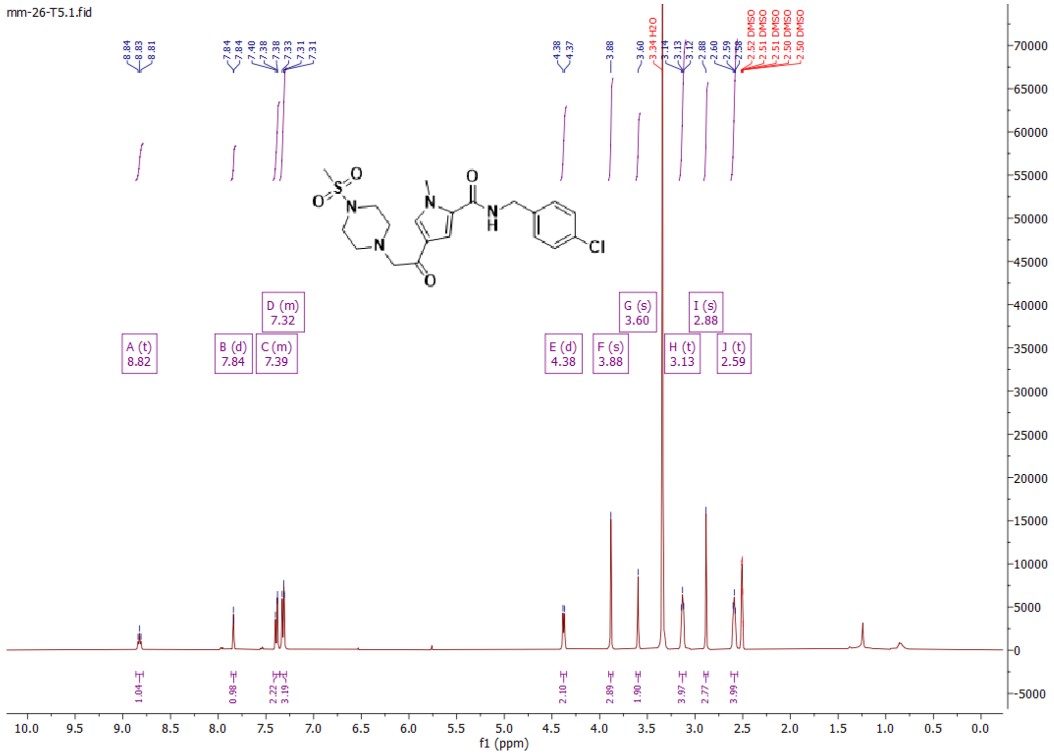

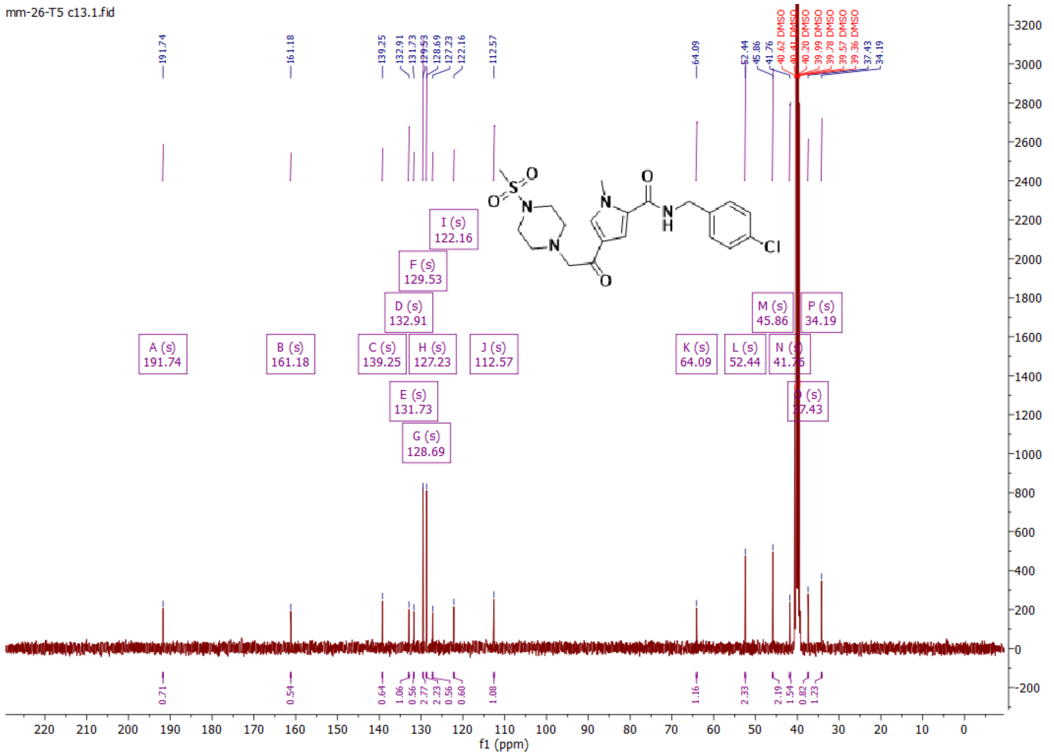

Supplement: Supplementary file 1 [file ao5c08248_si_001.pdf]
